# TAK1 inhibition activates pore-forming proteins to block intracellular bacterial growth through modulating mitochondria

**DOI:** 10.1038/s41419-025-07760-4

**Published:** 2025-06-18

**Authors:** Wilfred López-Pérez, Roland E. González-Calderón, Kazuhito Sai, Prashant Rai, Jacqueline M. MacStudy, Yosuke Sakamachi, Cameron Parsons, Sophia Kathariou, Michael B. Fessler, Jun Ninomiya-Tsuji

**Affiliations:** 1 Department of Biological Sciences and Toxicology Program, North Carolina State University, Raleigh, NC USA; 2 Immunity, Inflammation and Disease Laboratory, National Institute of Environmental Health Sciences, National Institute of Health, Research Triangle Park, NC USA; 3 Department of Food, Bioprocessing, and Nutrition Sciences, North Carolina State University, Raleigh, NC USA

**Keywords:** Cell death, Innate immune cells

## Abstract

Mitogen-activated protein kinase kinase kinase 7 (MAP3K7), known as TAK1, is a central mediator of intracellular host defense signaling promoting inflammatory gene expression. Hence, TAK1 is a prime target of intracellular bacterial effectors in blocking inflammatory responses. However, when TAK1 is inhibited, host cells alternatively activate multiple cell death pathways, namely caspase 8-dependent apoptosis and pyroptosis, and receptor interacting protein kinase 3 (RIPK3)-dependent necroptosis. While these pathways ultimately lead to cell death, we found that they also modulate mitochondria to produce mitochondrial reactive oxygen species (ROS). Although as cell death executors, mixed lineage kinase-like (MLKL) and gasdermins are known to form pores in the plasma membrane, we found that TAK1 inhibition translocates them to mitochondria resulting in elevated mitochondrial ROS. Ablation of both MLKL and gasdermins diminished TAK1 inhibition-induced elevation of ROS and exacerbated intracellular bacterial colonization. Our results reveal that these cell death pathways have an alternative host defense role to prevent intracellular pathogen colonization.

## Introduction

Bacterial invasion initiates host inflammatory signaling pathways, which include pathways leading to canonical inflammatory responses and several different types of cell death, apoptosis, pyroptosis, and necroptosis [1]. An initiator caspase, caspase 8, mediates apoptosis and pyroptosis, while activation of receptor interacting protein kinase 3 (RIPK3) leads to necroptosis. Mitogen-activated protein kinase (MAPK) kinase kinase 7, known as TAK1, is one of the central signaling intermediates in the canonical inflammatory pathway [2]. TAK1 transmits the Toll like receptors and TNF signals to transcription factors, NF-κB and AP-1, through activating IKKs and MAPKs, respectively. In characterizing murine tissues and cultured cells harboring *Tak1* gene deletion, we have found that *Tak1* ablation abolishes the NF-κB and MAPK-driven inflammatory pathway but activates cell death pathways, which causes severe inflammatory conditions [3, 4]. Thus, both activation and inhibition of TAK1 results in inflammation, which had long been puzzling to us. Pathogens express a variety of effector molecules to block host inflammatory signaling pathways [5] including TAK1 [6]. Such efforts are supposed to benefit pathogens. However, we have recently found that the TAK1 inhibition-induced caspase 8 and RIPK3 cell death pathways elevate mitochondrial reactive oxygen species (ROS), which ultimately block intracellular pathogen growth before killing host cells [7]. We proposed that TAK1 inhibition-induced cell death pathways are alternative host defenses that have evolved to counteract the effects of pathogens’ effectors [7]. The current study aimed to define TAK1 inhibition-induced alternative host defense mechanism. Specifically, we determined the mechanism by which caspase 8 and RIPK3 induce mitochondrial ROS upon TAK1 inhibition.

## Results

### Compound deletion of caspase 8 and *Mlkl* blocks *Tak*1 deficiency-induced ROS and cell death

**Fig. 1:**
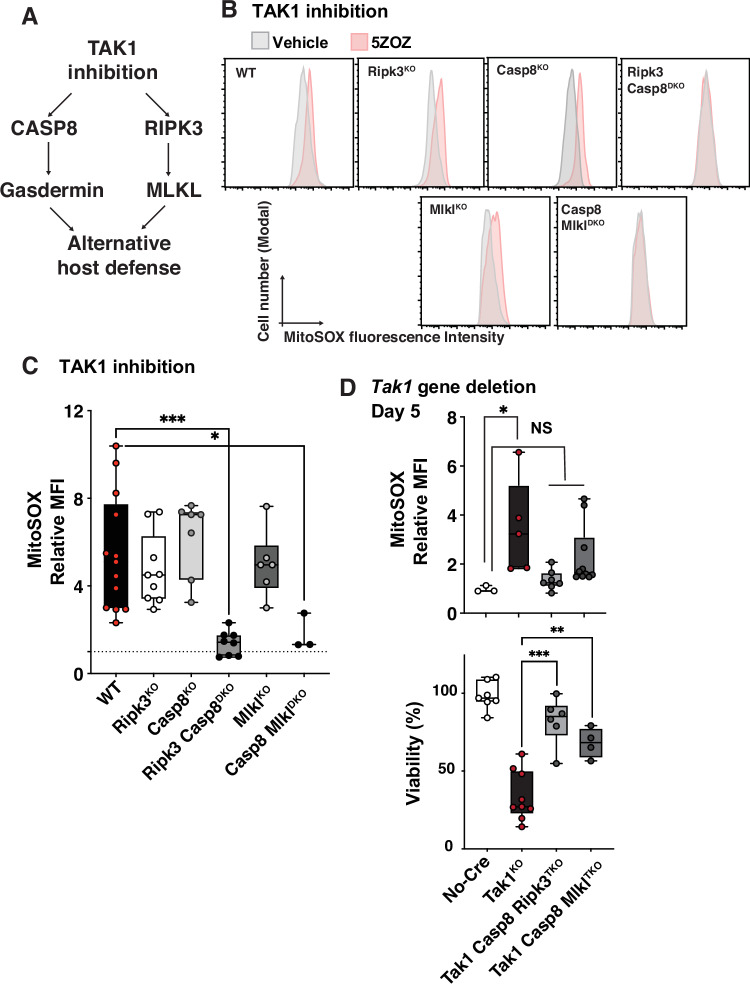
Compound deletion of caspase 8 and *Mlkl* blocks *Tak*1 deficiency-induced ROS and cell death. **A** Working hypothesis. We hypothesize that TAK1 inhibition-induced pathways block intracellular bacteria colonization through pore-forming proteins, MLKL and gasdermins. **B**, **C** Caspase 8-deficient and Caspase 8 and *Mlkl* double-deficient BMDMs were generated by treating BMDMs isolated from mice harboring inducible *Casp8* deletion (*Rosa26CreERT Casp8*^*flox/flox*^) and inducible *Casp8* and whole body *Mlkl* deletions (*Rosa26CreERT Casp8*^*flox/flox*^*, Mlkl*^*−/−*^) with the Cre inducer, 4OHT, for 5 days. Other BMDMs were isolated from the mice harboring indicated genotypes. All BMDMs were treated with vehicle (DMSO) or 300 nM 5z-7oxozeanol (5ZOZ) for 18 h. SytoxGreen-negative (live) BMDMs were gated and analyzed. Representative MitoSOX analyses were shown in (**B**) and quantification of replicate experiments is shown in (**C**). **D** BMDMs were isolated from mice harboring inducible *Tak1* gene deletion (*Rosa26CreERT Tak1*^*flox/flox*^) alone or together with other gene deletions, and were incubated with 4OHT for 5 days. MitoSOX analysis and crystal violet cell viability were performed. Median fluorescence intensity (MFI) in live BMDMs and crystal violet absorbance relative to vehicle treated BMDMs are shown. One-way ANOVA, multiple comparisons, Tukey test; ***, *p* < 0.001; **, *p* < 0.01; *, *p* < 0.05; NS not significant.

*Tak1* gene deletion or pharmacological inhibition of TAK1’s protein kinase activity elevates ROS, which ultimately kill the cells [2]. This ROS-cell killing requires inflammatory cytokine TNF stimulation in many cell lines and primary keratinocytes [8–10]. However, in bone marrow derived macrophages (BMDMs), *Tak1* gene deletion or TAK1 inhibition alone can elevate ROS and cell death without additional stimuli [7, 11], because BMDMs produce TNF. We previously reported that double deletion of caspase 8 and *Ripk3* but no single deletion blocked *Tak1* deficiency-induced mitochondrial ROS in BMDMs [7]. Thus, both caspase 8 and RIPK3 pathways are involved in elicitation of mitochondrial ROS. Many studies have determined the effector molecules downstream of caspase 8 and RIPK3 [12]. Caspase 8 initiates the caspase cascade leading to apoptosis, but also cleaves and activates gasdermin D (GSDMD) [13]. RIPK3 phosphorylates and activates mixed lineage kinase-like (MLKL) [14]. Both GSDMD and MLKL are known to be oligomerized and form pores in the cytoplasmic membrane, which execute pyroptosis and necroptosis, respectively. While they preferentially bind to phospholipids particularly phosphatidylinositol in the cytoplasmic membrane, they are also known to bind to cardiolipin [15–17], which is abundant in the mitochondrial inner membranes. Several earlier studies reported that gasdermins and MLKL modulate mitochondria [18–21]. We hypothesized that GSDMD and MLKL participate in elicitation of mitochondrial ROS in TAK1 ablation (Fig. 1A). We began with investigating involvement of MLKL by using *Mlkl* deficient mice [22]. We have previously reported that TAK1 inhibition with two different pharmacological inhibitors, 5Z-7oxozeaenol (5ZOZ) [23] and Takinib [24] as well as *Tak1* gene deletion-induced ROS [7]. These ROS can be detected with two different mitochondrial specific ROS dyes, MitoSOX and MitoPY1, but ineffectively with general ROS dye CM-H2DCFDA. The ROS signals colocalize with mitochondria [7]. This ROS elevation begins around 5–6 h post TAK1 inhibition and is observed at a high level after 18 h when BMDMs with TAK1 inhibition are still similarly viable compared with no treatment [7]. Thus, in the current study, we examined the MitoSOX levels at 18 h post TAK1 inhibition, when TAK1 inhibition did not reduce cell viability compared with non-treated BMDMs (Supplementary Fig. S1). Consistent with our previous results [7], TAK1 inhibition alone with the TAK1 inhibitor 5Z-7oxozeaenol (5ZOZ) elevated mitochondrial ROS in BMDMs, which were abolished by double gene deletion of caspase 8 and *Ripk3* (Fig. 1B, C). *Mlkl* deficiency did not block TAK1 inhibition-induced mitochondrial ROS (Fig. 1B, C). However, macrophages harboring double deletions of caspase 8 and *Mlkl* exhibited diminished TAK1 inhibition-induced mitochondrial ROS. Double deletions of caspase 8 and *Mlkl’*s effectiveness in diminishing ROS was slightly less or comparable to caspase 8 and *Ripk3* double deficiency (Fig. 1B, C). When floxed *Tak1* gene was deleted by inducible Cre recombinase (CreERT) expression, TAK1 protein was decreased, mitochondrial ROS increased, and BMDMs started dying at 5 days (Supplementary Fig. S1B and Fig. 1D). We observed that caspase 8 and RIPK3 protein levels also decreased compared with the loading control as cells started dying (Supplementary Fig. S1B, lane 2), which may be partially due to caspase 8 activation (self-cleavage) and RIPK3 degradation [25]. At 8 days post-*Tak1* gene deletion, BMDMs were almost completely killed (Supplementary Fig. S1C, D). Compound inducible deletion of *Caspase 8* and germline *Ripk3* deletion but not any single deletion reduced *Tak1* deficiency-induced mitochondrial ROS and cell death (Fig. 1D and Supplementary Fig. S1D). We examined whether caspase 8 and *Mlkl* double deletion rescued *Tak1* gene deletion-induced mitochondrial ROS and cell death, The rescues were highly noticeabe but not complete (Fig. 1D). Overall, caspase 8 and *Mlkl* double deletions was similarly effective compared with caspase 8 and *Ripk3* double deletion. These results demonstrate that MLKL is the major mediator of RIPK3-dependent mitochondrial ROS and subsequent cell death.

### Gasdermins participate in TAK1 inhibition-induced mitochondrial ROS

**Fig. 2:**
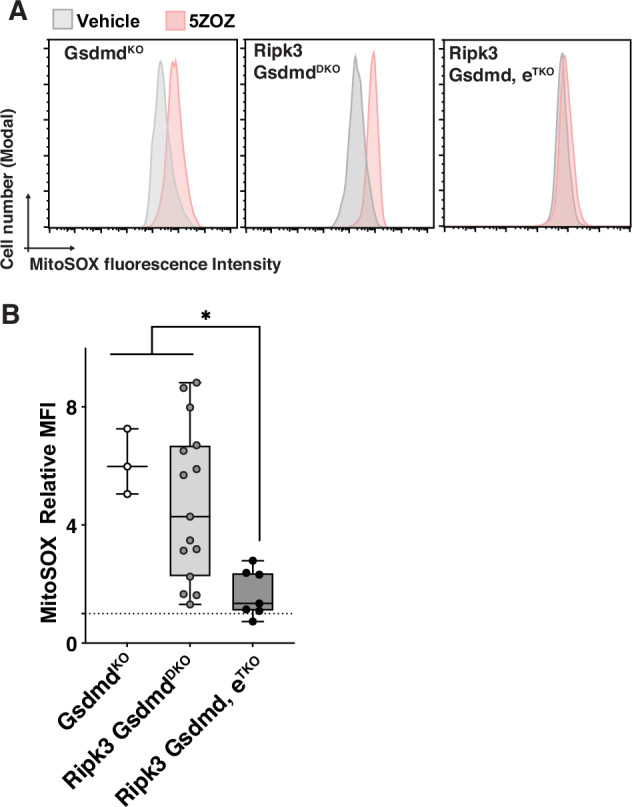
Gasdermins are downstream effector of caspase 8 in TAK1 inhibition-induced mitochondrial ROS. **A**, **B** BMDMs isolated from the indicated genotypes were treated with 300 nM 5ZOZ for 18 h. Representative MitoSox analysis in live BMDMs is shown (**A**). Median fluorescence intensity (MFI) in live BMDMs relative to vehicle treated BMDMs is shown (**B**). One-way ANOVA, multiple comparisons, Tukey test; *, *p* < 0.05.

We next asked whether gasdermin D (GSDMD), which is known to play a major role downstream of caspase 8 [26, 27], participates in elicitation of mitochondrial ROS by using BMDMs with *Gsdmd* and *Ripk3* gene deletion. These BMDMs were still viable by TAK1 inhibition at 18 h at a similar level to non-treated BMDMs (Supplementary Fig. S2A). Gene deletion of *Gsdmd* [28] alone did not block TAK1 inhibition-induced mitochondrial ROS (Fig. 2A, B). We generated mice harboring *Ripk3* and *Gsdmd* double deletion and tested the TAK1 inhibition in BMDMs. However, unlike *Caspase8 Mlkl* double deletion, *Ripk3* and *Gsdmd* double deletion did not effectively block TAK1 inhibition-induced mitochondrial ROS (Fig. 2A, B). Gasdermins are a protein family [13, 29], and multiple members are known to be expressed in macrophages including gasdermin E (GSDME) [18]. To test whether gasdermin E is involved in TAK1 inhibition-induced mitochondrial ROS, we used BMDMs from *Ripk3*, *Gsdmd* and *Gsdme* triple deletion mice. *Ripk3*, *Gsdmd* and *Gsdme* triple deletion inhibited TAK1 inhibition-induced mitochondrial ROS (Fig. 2A, B), suggesting that both GSDMD and GSDME participate in mitochondrial ROS elevation. Likewise, gasdermin inhibitor disulfiram [30], which can inhibit not only GSDMD but also GSDME [31], inhibited TAK1 inhibition-induced mitochondrial ROS in *Ripk3*-deficient BMDMs (Supplementary Fig. S2B). These results demonstrate that two types of pore forming proteins, MLKL and gasdermins, mediate TAK1 inhibition-induced mitochondrial ROS.

### Intracellular *Salmonella* growth suppression is dependent on MLKL and gasdermins

**Fig. 3:**
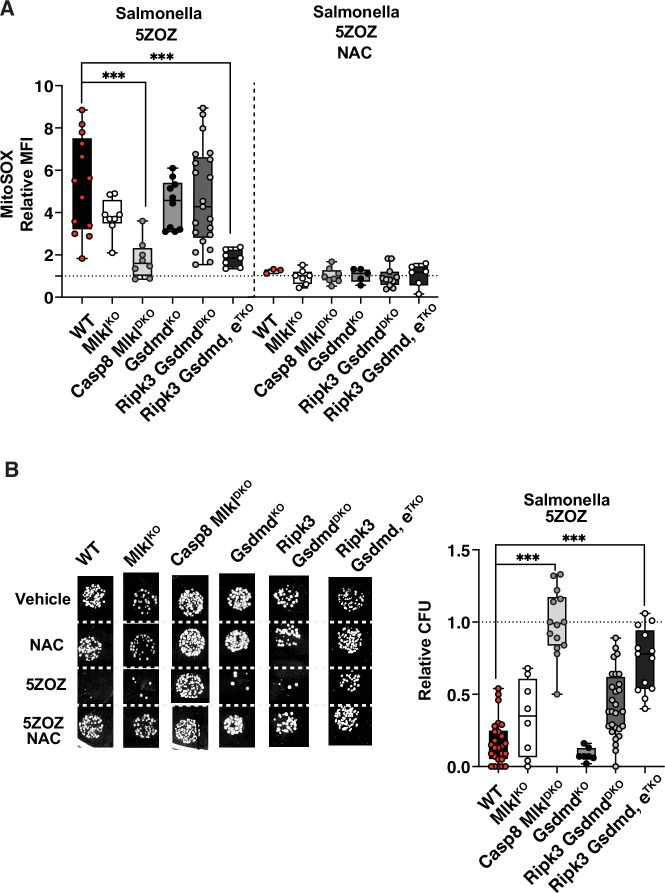
Intracellular *Salmonella* colonization. **A** BMDMs isolated from the indicated genotypes were infected with *Salmonella* (MOI, 10) for 30 min, and extracellular *Salmonella* was eliminated by gentamicin treatment. BMDMs treated with 300 nM 5ZOZ alone or together with 3 mM N-acetyl cysteine (NAC) for 18 h. MitoSOX MFIs in live BMDMs relative to samples with *Salmonella* infection alone are shown. *Salmonella* infection alone or with NAC did not elevate mitochondrial ROS (Supplementary Fig. S3B). **B** Intracellular *Salmonella* colonization was assessed at 18 h post infection with and without 300 nM 5ZOZ and/or 3 mM NAC. Representative *Salmonella* colony photos are shown (left). Each column of colony photos are from one agar plate. Colony forming units (CFUs) relative to vehicle treatment are shown (right). One-way ANOVA, multiple comparisons, Tukey test; ***, *p* < 0.001; **, *p* < 0.01; *, *p* < 0.05; NS not significant.

We reported that TAK1 inhibition-induced mitochondrial ROS is associated with blockade of intracellular bacterial colonization [7]. We asked whether MLKL and gasdermins mediate this bactericidal activity. We used *Salmonella enterica serovar* Typhimurium LT-2, hereafter referred to as *Salmonella*, as a model intracellular bacterial strain. While certain intracellular bacteria, such as *Yersinia* genus, express an effector directly inhibiting TAK1 enzymatic activity [6], *Salmonella* does not inhibit TAK1 [32]. Thus, we can examine the effect of TAK1 inhibition in intracellular bacterial growth with and without TAK1 inhibitor. We incubated BMDMs with *Salmonella* (multiplicity of infection (MOI), 10), and at 30 min post-infection, extracellular *Salmonella* were eliminated by gentamicin, and TAK1 inhibitor 5ZOZ were added. As *Salmonella* LT-2 strain is non-virulent in mice [33], it does not cause cell lysis in BMDMs. At 18 h post-infection, BMDMs are similarly viable with and without TAK1 inhibition (Supplementary Fig. S3A). We measured the mitochondrial ROS levels in live BMDMs and the intracellular *Salmonella* numbers (Fig. 3). *Salmonella* infection alone did not affect mitochondrial ROS, which is consistent with the notion that *Salmonella* does not inhibit TAK1 (Supplementary Fig. S3B). *Mlkl* or *Gsdmd* single or *Ripk3 Gsdmd* double deletion did not effectively block TAK1 inhibitor-induced mitochondrial ROS, whereas double deletion of caspase 8 and *Mlkl* or triple deletion of Ripk3, *Gsdmd* and *Gsdme* was effective on blocking it (Fig. 3A, left). We previously reported that hydrogen sulfide, cysteine, and N-acetyl cysteine (NAC), all of which can provide electrons to the mitochondrial electron transport chain [34], completely block bacteria growth suppression by TAK1 inhibition, whereas a general ROS scavenger, tert-butylhydroquinone, is ineffective [7]. Thus, ROS by themselves are not sufficient, but other events associated with disruption mitochondrial respiration are also important to block intracellular bacteria growth. We also reported that NAC exacerbates intracellular colonization of *Yersinia* that expresses a TAK1 inhibiting effector YopJ [7]. Therefore, in this study, we used NAC treatment as a control for restoration of TAK1 inhibitor-induced events. NAC abolished TAK1 inhibition-induced mitochondrial ROS in BMDMs with any gene deletion (Fig. 3A, right). The intracellular bacteria numbers were reduced by TAK1 inhibition, which were restored by caspase 8 and *Mlkl* double deletion or *Ripk3, Gsdmd,* and *Gsdme* triple deletion but not by *Mlkl* or *Gsdmd* single deletion or *Ripk3 Gsdmd* double deletion (Fig. 3B). NAC restored intracellular *Salmonella* numbers in all genotypes (Fig. 3B and Supplementary Fig. S3C). These results demonstrate that MLKL and gasdermins (GSDMD and GSDME) cooperatively promote blockade of intracellular *Salmonella* colonization through mitochondrial modulation.

### Mitochondrial localization of MLKL and gasdermins upon TAK1 inhibition

**Fig. 4:**
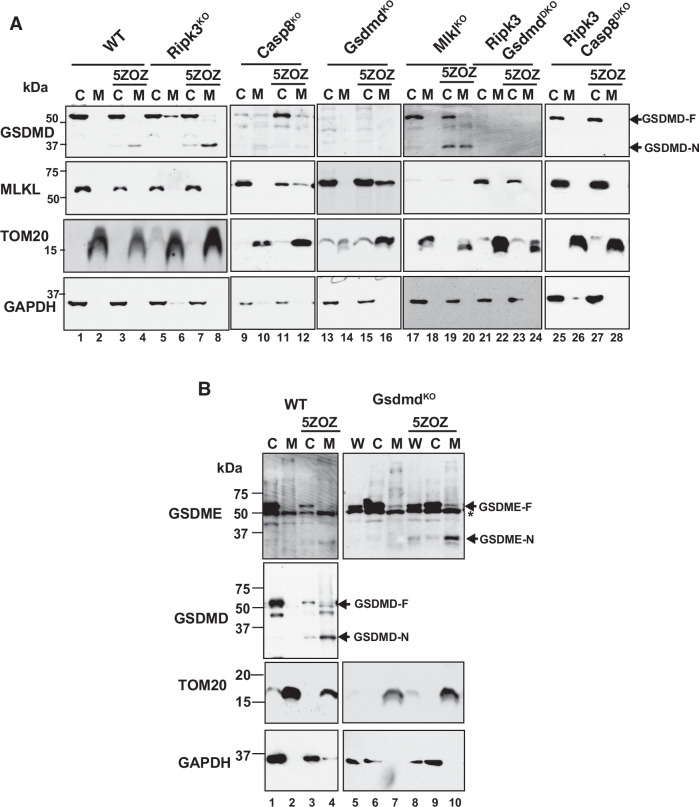
Mitochondrial localization of gasdermins and MLKL. **A** BMDMs were treated with vehicle or 300 nM 5ZOX for 5 h. Cell lysates were fractionated into the cytosol including plasma membrane (C) and the mitochondrial (M) fractions, and GSDMD and MLKL were analyzed by Western blotting. Full length GSDMD (GSDMD-F) and N-terminal GSDMD (GSDMD-N) are indicated. **B** GSDME and GSDMD in the whole cell lysate (W), the cytosol including plasma membrane (C) and the mitochondrial (M) fractions in wild type (left panels) and *Gsdmd*-deficient BMDMs with vehicle or 300 nM 5ZOZ (5 h) were analyzed by Western blotting. Full length GSDME (GSDME-F) and N-terminal GSDME (GSDME-N) are also indicated. *, non-specific band. Mitochondrial marker TOM20 and cytosolic marker GAPDH are shown as loading controls.

**Fig. 5:**
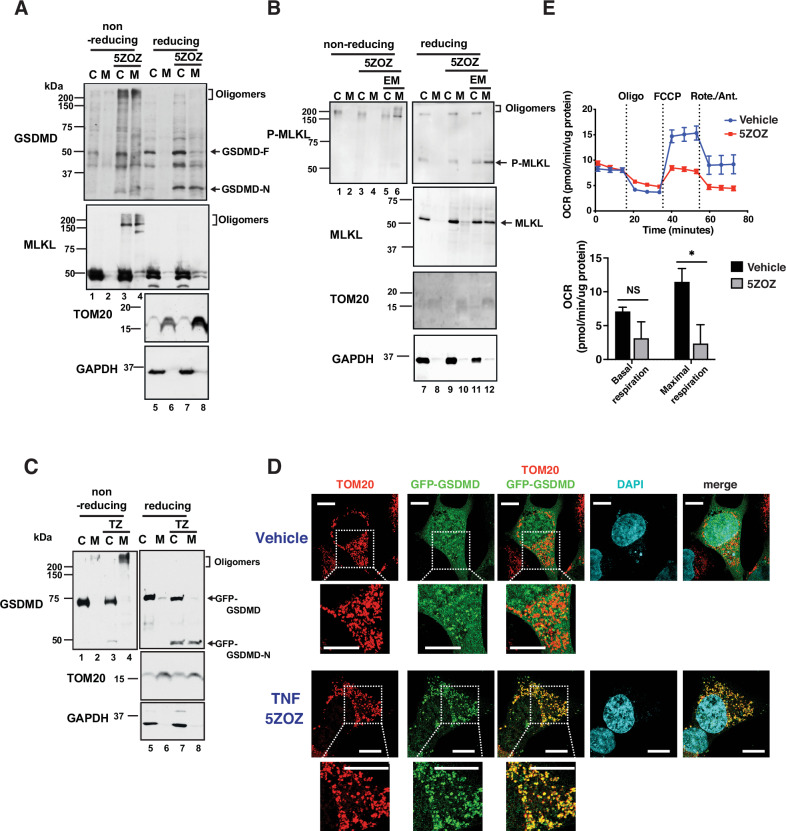
Gasdermins and MLKL disrupt mitochondria. **A**, **B** Wild type BMDMs were treated with vehicle or 300 nM 5ZOZ for 5 h. The cytosolic including plasma membrane (C) and the mitochondrial (M) proteins were resolved by SDS-PAGE with and without a reducing agent (2-mercaptoehtanol), and analyzed by Western blotting. Oligomerized bands of GSDMD and MLKL and N-terminal GSDME are indicated. Mitochondrial marker TOM20 and cytosolic marker GAPDH are shown as loading controls. **B** 20 µM pan-caspase inhibitor, emricasan, was also treated for 5 h. Smaller amounts of proteins were loaded to avoid anti-P-MLKL background bands. **C** HeLa-RIPK3 cells were transfected with vectors expressing GFP alone or GFP-GSDMD. At 24 h post-transfection, cells were treated with vehicle or 50 ng/ml TNF and 1 μM 5ZOZ (TZ) for 5 h. Cytosol and mitochondrial fractions were analyzed in reducing and non-reducing conditions by Western blotting. TOM20 and GAPDH are shown as loading controls for mitochondria and cytosol, respectively. The estimated molecular weights of GFP-GSDMD and cleaved GFP-GSDMD-N are about 75 and 50 kDa, respectively. **D** HeLa-RIPK3 with GFP-GSDMD were treated with vehicle (top and 2nd panels) or with 50 ng/ml TNF and 1 µM 5ZOZ (3rd and bottom panels) for 5 h. Immunofluorescence staining of anti-TOM20 (red) and GFP-GSDMD (green) was analyzed by confocal microscopy. 2nd and bottom panels show enlarged images of top and 3rd panels, respectively. Scale bars, 10 µm. Control GFP expressing cells are shown in Supplementary Fig. S5A. Volumetric (3D) image of the TNF and 5ZOZ treated cell are shown in Supplementary Video 1. **E** Wild type BMDMs were treated with vehicle or 300 nM 5ZOZ for 4 h. Mitochondrial respiration was determined by Seahorse assay (Oligo, oligomycin; FCCP, carbonyl cyanide 4-(trifluoromethoxy) phenylhydrazone; Rote/Ant, rotenone + antimycin A). A representative analysis is shown (upper panel). Oxygen consumption rate (OCR) was normalized with protein concentration per well (lower graph). Bars represent mean values of basal respiration and maximal respiration from three independent Seahorse assays. Students’ *t*-test, ***, *p* < 0.001; **, *p* < 0.01; *, *p* < 0.05; NS not significant.

We next examined whether MLKL and gasdermins are physically associated with mitochondria upon TAK1 inhibition. If MLKL and GSDMD modulate mitochondria to produce ROS, they should translocate to mitochondria prior to mitochondrial ROS elevation. We isolated cytosolic (C), including the plasma membrane, as well as mitochondrial (M) fractions at 5 h post-TAK1 inhibition. Without TAK1 inhibition, GSDMD and MLKL were found to reside in the cytosol (Fig. 4A, top and 2nd panels) [35]. N-terminus GSDMD (GSDMD-N), which forms a pore [13, 29], was observed both in the cytosolic and mitochondrial fractions with TAK1 inhibition in wild type BMDMs (Fig. 4A, top panel, lanes 3, 4). MLKL was not detectable in the mitochondrial fraction (2nd panel, lane 4). These suggest that GSDMD-N physically translocated to the mitochondria upon TAK1 inhibition. *Ripk3* deletion did not affect GSDMD-N translocation to the mitochondrial fraction (top panel, lane 8). In caspase 8-deficient BMDMs, we observed increased MLKL in the mitochondria with TAK1 inhibition (2nd panel, lane 12), whereas GSDMD cleavage was marginal (top panel, lanes 11 and 12). These are consistent with the previous notion that caspase 8 is the direct mediator of GSDMD cleavage upon bacterial TAK1 inhibition [26, 27]. Single deletion of *Gsdmd* seemed to exacerbate mitochondrial translocation of MLKL (2nd panel, lane 16). We also found that mitochondrial MLKL was detectable with S345 phospho-specific MLKL antibody in wild type BMDMs with pan-caspase inhibition (see Fig. 5B, lane 12), indicating that MLKL is activated for pore formation. Single deletion of *Mlkl* induced mitochondrial translocation of GSDMD (top panel, lane 20) at the level comparable to that in wild type (lane 4). Double deletion of *Ripk3* and *Gsdmd* or caspase 8 abolished both GSDMD and MLKL translocation to the mitochondria (lanes 21–28). We also found that another TAK1 inhibitor Takinib induces GSDMD translocation to the mitochondria (Supplementary Fig. S4A). These results demonstrate that either or both GSDMD-N or/and phosphorylated MLKL are translocated to mitochondria in response to TAK1 inhibition depending on availability of caspase 8-GSDMD and RIPK3-MLKL pathways. If one pathway is disrupted, the other pore forming proteins translocate to mitochondria upon TAK1 inhibition. Given barely detectable MLKL translocation in wild type and its exacerbation in *Gsdmd*-deficient BMDMs, GSDMD may be a primary mediator of TAK1 inhibition-induced mitochondrial modulation. Our results in Figs. 2 and 3 indicate that double deletion of *Ripk3* and *Gsdmd* is insufficient, but that triple deletion of *Ripk3*, *Gsdmd, and Gsdme* can block TAK1 inhibition-induced mitochondrial ROS (Fig. 2B). Like GSDMD, GSDME is also cleaved by caspases and the resulting N-terminal GSDME (GSDME-N) forms a pore [13, 29]. We examined GSDME cleavage and its localization in wild type, *Gsdmd* single*- and Ripk3 and Gsdmd* double*-*deficient BMDMs (Fig. 4B and Supplementary Fig. S4B). GSDME-N was found to be in the mitochondrial fraction in *Gsdmd*- and *Ripk3 and Gsdmd double-*deficient BMDMs when TAK1 was inhibited, whereas the GSDME-N was only marginally observed in wild type BMDMs (Fig. 4B, lanes 4, 10, Supplementary Fig. S4B, lanes 4, 13). Consistent with a previous study [31], disulfiram blocked cleavage of GSDME as well as GSDMD (Supplementary Fig. S3B, lanes, 5,6,10,14). These results suggest that GSDME plays a compensatory role when GSDMD is absent.

Both MLKL and N-terminal gasdermins form pores by oligomerization [13, 29]. We examined whether TAK1 inhibition induces MLKL and gasdermin oligomerization in the mitochondria. In non-reducing SDS-PAGEs, GSDMD were observed as high molecular weight smear bands with TAK1 inhibition in the both cytosolic and mitochondrial fractions of wild type BMDMs (Fig. 5A top panel, lanes 3, 4). A higher molecular weight MLKL was observed when Western blotting was overexposed (Fig. 5A 2nd panel, lanes 3, 4). RIPK3 phosphorylates MLKL at serine 352, which promotes MLKL oligomerization [22]. Phosphorylated MLKL was clearly detected in the mitochondrial fraction as smear bands at high molecular weights with TAK1 inhibition when pan-caspase inhibitor, emricasan, was treated (Fig. 5B top panel, lane 6). These suggest that TAK1 inhibition primarily induces N-terminal GSDMD oligomerization in mitochondria, and phosphorylation and oligomerization of MLKL occurs when caspases are inhibited. To visualize physical location of GSDMD, we used GFP-tagged GSDMD. GFP-tagged GSDMD was expressed in RIPK3 expressing HeLa (HeLa-RIPK3) cells, in which we previously reported that co-treatment of TNF and TAK1 inhibitor mimics pathogen infection and induces mitochondrial ROS [7]. Although N-terminal tag is known to disrupt functional pore formation [36], we were able to observe cleaved GFP-GSDMD and oligomerization in the mitochondrial fraction at 5 h post-TNF and TAK1 inhibitor treatment (Fig. 5C). Analysis via confocal microscopy indicated that the GFP-GSDMD fusion protein was dispersed within the cell (Fig. 5D, upper panes), and GFP-GSDMD puncta were formed in the cytoplasm following TNF and TAK1 inhibitor treatment, which were overlapped with mitochondria (Fig. 5D, lower panels, Supplementary Fig. S5B and Supplementary Video 1). Control cells expressing only GFP lacked specific colocalization with mitochondria (Supplementary Fig. S5A, B and Supplementary Video 2). Live cell time lapse images also demonstrate co-localization of GFP-GSDMD with mitochondria starting around 5 h post stimulation (Supplementary Fig. S5C). This co-localization was also observed in cells with Salmonella (Supplementary Fig. S5D). Thus, GFP-GSDMD is likely to be cleaved and localized to mitochondria under pathogen infection. Finally, we found that mitochondrial respiration was disrupted with TAK1 inhibition (Fig. 5E). Mitochondrial impairment was also observed as a lower level of the mitochondrial membrane potential with TAK1 inhibition (Supplementary Fig. S5E). Mitochondrial respiration and membrane potential were partially restored by *Ripk3*, *Gsdmd,* and *Gsdme* triple deletion (Supplementary Fig. S5E, F). Collectively, these results demonstrate that gasdermins, GSDMD and GSDME, and/or MLKL are translocated to mitochondria upon TAK1 inhibition, which disrupt mitochondrial respiration resulting in elevation of mitochondrial ROS and blocking intracellular *Salmonella* colonization (Graphic summary, Supplementary Fig. S6).

## Discussion

In the current study, we investigated how host cells prevent intracellular bacteria growth through mitochondrial modulation when TAK1 is inhibited. We found that, as initially hypothesized (Fig. 1A), both caspase 8-GSDMD and RIPK3-MLKL pathways cooperatively modulate mitochondria. We found that their cell death executor pore forming proteins, MLKL and GSDMD, are translocated to mitochondria, and that GSDME plays a compensatory role when GSDMD is absent. We previously reported that hydrogen sulfide and its precursors, NAC, but not general antioxidants effectively block TAK1 inhibition-induced mitochondrial ROS [7]. As hydrogen sulfide facilitates mitochondrial respiration by donating electrons to complex III in the respiratory chain, TAK1 inhibition is likely to promote ROS production by disrupting the electron flux in the respiratory chain. Our current study identified that TAK1 inhibition indeed impairs mitochondrial respiration, and that RIPK3-MLKL and gasdermins are the executors of this disruption. How do these proteins disrupt mitochondrial respiration? Both MLKL and gasdermins are known to bind particular membrane phospholipids such as phosphatidylinositol to form pores, but also exhibit a high affinity to cardiolipin [15–17]. Cardiolipin is abundant in the mitochondrial inner membrane where the respiratory chains reside. Thus, it may be reasonable to assume that MLKL and gasdermins form pores in the mitochondrial inner membrane, which results in disrupting the membrane potential leading to blocking the electron flux. However, how these proteins reach the mitochondrial inner membrane is still elusive as the outer mitochondrial membrane is impermeable to them under normal conditions. Rogers et al. reported that N-terminus GSDME has an ability to permeabilize the outer mitochondrial membrane [18]. Caspase 8 cleaves and activates BH3-only protein Bid, and cleaved Bid (tBid) might promote the canonical apoptotic outer membrane pores of BAX and BAK [37], which could permeabilize outer mitochondrial membrane to MLKL and gasdermins. Alternatively, cardiolipin translocation to outer mitochondrial membrane is known to occur under apoptotic conditions [38] and pyroptosis [39], which might recruit MLKL and gasdermins and permeabilize outer mitochondria membrane. These mechanisms may be cooperatively accountable for disrupting the mitochondrial outer membrane in BMDMs with TAK1 inhibition.

*Tak1* gene deletion causes cell death in many types of cells and tissues [2]. We initially thought that TAK1 is required for cell survival. However, it turns out that artificial deletion of *Tak1* gene mimics pathogen effectors that target host inflammatory responses. Specifically, *Yersinia* species including *Y. pseudotuberculosis*, *Y. enterocolitica*, and *Y. pestis*, express an effector YopJ, an acetyl transferase, which directly modifies the amino acid residue (Thr-187) of TAK1’s activation loop disrupting the protein kinase catalytic activity [6]. Once TAK1 is inhibited or ablated, host cells initiate the alternative defense mechanisms involving cell death pathways. Our current results indicate that such inhibition initiates activation of MLKL and gasdermins to block pathogen colonization. Besides TAK1, many other protein kinases in the inflammatory signaling pathways are the targets of bacterial and viral effectors [5], which may be covalently modified or degraded [40]. For example, IκB kinases (IKKs), activators of NF-κB, and TANK binding kinase 1 (TBK1), an activator of the IRF-interferon pathway, can be inhibited upon pathogen invasion. Interestingly, similar to *Tak1* ablation [2], mice harboring *Ikk* or *Tbk1* gene deletion exhibit tissue damage associated with cell death [41, 42]. The severity of tissue damage varies dependent on tissues and genes. Nevertheless, both caspase 8 and RIPK3 pathways appear to be involved in *Tak1*-, *Ikk*-, and *Tbk1*-deficient cell death. This might suggest that a common mechanism mediates the alternative host defense when inflammatory protein kinases are inhibited by pathogen effectors.

The limitation of our current results lies in the absence of identification of the mechanism by which TAK1 inhibition initiates the caspase 8 and RIPK3 cell death pathways. Receptor interacting protein kinase 1 (RIPK1) has been reported as a substrate of TAK1, IKKs, and TBK1 [43–46]. Their phosphorylation limits RIPK1 activity resulting in blockade of caspase 8 and RIPK3. Thus, RIPK1 may serve as a common sensor for pathogen effectors’ inhibition of inflammatory protein kinases. However, RIPK1 alone cannot be accountable for TAK1 ablation-induced cell death [47]. Another limitation of this study is that the triple deletion of *Ripk3*, *Gsdmd*, and *Gsdme* largely, but not completely, blocks mitochondrial ROS and bacterial growth suppression by TAK1 inhibition (Fig. 3). This indicates that additional mechanisms are involved in TAK1 inhibition-induced mitochondrial ROS elevation. Other gasdermins may be involved, although their expression levels are much lower than those of GSDMD and GSDME in macrophages [29]. Apoptotic mitochondrial pore-forming proteins BAX/BAK are activated by caspase 8 through caspase 3. With BAX/BAK pores, it is reported that caspase 3 translocates into the mitochondria and disrupts the mitochondrial electron transport chain [48]. These possibilities need to be evaluated.

MLKL and gasdermins were originally described as cell death executors that form pores in the plasma membranes [13, 29]. However, emerging studies have been revealing that they also play other roles independently of executing cell death. MLKL binds to intracellular *Listeria* and blocks their colonization without inducing host cell death [49]. Gasdermin pores release interleukin 1 without largely disrupting the plasma membrane when the formation of gasdermin pores are limited [50]. Gasdermins are also reported to attack gram negative bacteria [51]. Our results demonstrate that MLKL and gasdermins target and disrupt mitochondria to produce ROS when TAK1 is inhibited by intracellular bacteria. All these functional roles of MLKL and gasdermins are associated with the host defense system. When MLKL and gasdermins are activated in response to invading microorganisms, the pore forming proteins may initially attempt to kill intracellular microorganisms by directly attacking them and/or activating inflammation by releasing IL-1. When microorganisms evade the initial host defense by inhibiting the canonical inflammatory signaling pathways, the pore forming proteins are translocated to and modulate mitochondria to initiate the bacteria-killing process. Finally, if the level of activated MLKL and gasdermins exceeds a certain threshold, they form pores in the host cell’s plasma membrane, resulting in host cell killing. Dead cells release danger associated molecular patterns, which promote inflammation in surrounding cells. MLKL and gasdermins may be the central mediators of this multi-layered host defense system.

## Materials and methods

### Study design

No power analysis for the sample size calculation was used. All experiments were conducted in BMDMs isolated from two-five different mice for each genotype. All independently acquired data points are shown in the results. The results are confirmed by at least three separately performed experiments. All methods were performed in accordance with the relevant guidelines and regulations.

### Mice, BMDMs, HeLa cells, and bacteria

As mice harboring germline *Tak1* or caspase 8 gene deletion are lethal during embryogenesis [2], we used an inducible gene deletion system. For other gene deletions, mice with germline gene deletions were used. Specifically, wild type [no-Cre *Tak1*^*flox/flox*^ [52]], inducible caspase 8-deficient [*Rosa26-CreERT*, JAX ® Mice, B6;129-*Gt(ROSA)26Sor*^*tm1(cre/ERT)Nat*^/J [53] *Casp8*^*flox/flox*^ [54]], *Ripk3*-deficient [*Ripk3*^*−/−*^ [55]], caspase 8 and *Ripk3* double deficient [*Casp8*^*−/−*^ (spontaneously acquired from *Casp8*^*flox/flox*^) *Ripk3*^*−/−*^], *Mlkl*-deficient [*Mlkl*^*−/−*^ [22]], *Gsdmd*-deficient [Gsdmd^−/−^, JAX ® Mice C57BL/6N-Gsdmdem4Fcw/J #032410 [28]], *Gsdme*-deficient [*Gsdmde*^*−/−*^, JAX ® Mice C57BL/6N-Gsdmeem1Fsha/J #032411 [56]], Casp8 *Mlkl* double-deficient (*Rosa26.CreERT Casp8*^*flox/flox*^ *Mlkl*^*−/−*^), *Gsdmd Ripk3* double-deficient (*Ripk3*^*−/−*^ *Gsdmd*^*−/−*^), *Ripk3, Gsdmd, Gsdme* triple-deficient (*Ripk3*^*−/−*^ *Gsdmd*^*−/−*^ *Gsdme*^*−/−*^), inducible *Tak1*-deficient (Rosa26-CreERT *Tak1*^*flox/flox*^), *Tak1 Casp8* double-deficient (*Rosa26.CreERT Tak1*^*flox/flox*^*, Casp8*^*flox/flox*^) *Tak1 Ripk3* double-deficient (*Rosa26.CreERT Tak1*^*flox/flox*^, *Ripk3*^*−/−*^), *Tak1, Casp8 and Ripk3* triple-deficient (*Rosa26.CreERT Tak1*^*flox/flox*^*, Casp8*^*flox/flox*^ *Ripk3*^*−/−*^) and littermate or age-matched no-Cre mice were used. The genotypes were determined by PCR and were confirmed by Western blotting. The mouse care and the procedures using mice were conducted with the approval of the North Carolina State University Institutional Animal Care and Use Committee.

Bone marrow cells were isolated with a standard method and were cultured in macrophage media; Dulbecco’s modified Eagle’s medium supplemented with 10% bovine growth serum (HyClone), 50 I.U./mL penicillin–streptomycin, and 30% L929 conditioned media at 37 °C with 5% CO_2_. For Cre-dependent gene deletion, BMDMs were treated with 0.3 μM 4-hydroxytamoxifen (4OHT) or vehicle (ethanol) alone for 5 days to achieve complete floxed gene deletion. To determine cell viability after gene deletions, BMDMs were fixed using 10% formalin in PBS, and stained with 0.1% crystal violet solution. The dye was eluted and analyzed at 595 nm. *Salmonella enterica* Typhimurium LT-2 were culture in Luria–Bertani (LB) broth at 30 °C.

### Reagents

TAK1 protein kinase inhibitor, 300 nM 5Z-7-oxozeaenol (5ZOZ) [23] and 10 µM Takinib [24] were used. 3 mM N-acetyl-L-cysteine (NAC) (Sigma-Aldrich), 50 μM disulfiram (Sigma-Aldrich) and pan-caspase inhibitor, emricasan, (MedKoo Biosciences, Inc.) were also used. To measure the level of ROS, 1.25 μM MitoSOX (Thermo Fisher Scientific), was used. To monitor cell viability, 30 nM Sytox Green (Thermo Fisher Scientific) was used. Antibodies against TAK1 [57], caspase 8 (CASP8) (Cell Signaling #4927), RIPK3 (Sigma-Aldrich, R4277 and MABC1595 clone 8G7), MLKL (Abcam, ab184718), phospho-MLKL (phospho S345) (Abcam, ab196436), gasdermin D (GSDMD) (Abcam, ab209845), gasdermin E (GSDME) (Abcam, ab215191), GFP (Abcam, ab1218), TOM20 (Santa Cruz, FL-145, Sigma-Aldrich, HPA011562), GAPDH (Chemicon, MAB374, Sigma-Aldrich, G9545), β-actin (Sigma-Aldrich, AC15) were used. Human TNF (Peprotech, 300-01 A), gentamicin sulfate (VWR), and 4OHT (Sigma-Aldrich) were also used.

### ROS analysis

BMDMs were washed with phosphate-buffered saline (PBS) and were detached from culture plates with gentle scraping. Cells were precipitated with centrifugation at 5000 × *g* for 1 min were incubated with mixtures of 1.25 μM MitoSOX and 30 nM Sytox Green in PBS for 30 min at room temperature. Live (Sytox green-negative) cells were gated, and the median fluorescence intensities (MFIs) were determined with a flow cytometer (Accuri C6 Plus, BD Bioscience) and FlowJo software (BC Bioscience).

### *Salmonella* infection

*Salmonella* were pre-cultured over-night, and were inoculated into fresh culture broth at 1:10 dilution. After 1.5 h-incubation at 30 °C, bacterial numbers were enumerated by OD, and bacteria were suspended in PBS after centrifugation at 5000 × *g* for 10 min. BMDMs were seeded on 6-well plates 1–3 days before infection. When cells reached to the confluency level at 80–100%, the culture medium was changed to one without any antibiotics, and the cell numbers per wells were counted. The cells were incubated with the indicated MOI of *Salmonella* for 30 min at 37 °C in 5% CO_2_. The cells were then washed with sterile PBS and incubated in a medium supplemented with gentamicin (100 μg/ml) for 18 h with the indicated chemicals. To determine the intracellular *Salmonella* growth, cells were washed with sterile PBS and lysed in PBS containing 0.2% Triton X-100 at 18 h-post infection. Serial dilutions of the lysates were spotted (10 μl/spot) on LB agar plates for enumeration of intracellular bacteria. For imaging, mCherry-Salmonella [7] were infected with the same procedure.

### Immunofluorescence Staining

To determine GSDMD localization, GFP-tagged GSDMD (pAcGFPC3-GSDMD) was generated using pAcGFPC3 (Takara) and mouse GSDMD [Addgene, Plasmid #80950 [51]]. HeLa cells stably expressing receptor interacting protein kinase 3 (HeLa-RIPK3) [49] were cultured with Dulbecco’s modified Eagle’s medium supplemented with 10% bovine growth serum (Hyclone) and were co-transfected with an empty GFP expressing vector or pAcGFPC3-GSDMD using TransIT-X2® (Mirus Bio). At 24 h post-transfection, cells were treated with 50 ng/ml TNF and 1 µM 5ZOZ. Some cells were used for live imaging with Mitotracker deep red (30 nM, Thermo Fisher) and Hoechst 33342 (1 µg/ml, Thermo Fisher) for 4–5 h by a confocal microscope (FV3000, Olympus) with a 40× oil objective (UPLXAPO40XO). Other cells were fixed with 4% paraformaldehyde for 10 min at room temperature, permeabilized with 0.5% Triton X100 for 10 min, and incubated with a blocking buffer, PBS containing 5% goat serum for 1 h at room temperature. Antibodies, anti-TOM20 (1:1000), and secondary antibodies, anti-rabbit IgG conjugated with Alexa-647 (1:1000, Thermo Fisher Scientific) were used. Cells were then counterstained with 4′,6-diamidino-2-phenylindole (DAPI) (Sigma-Aldrich). The coverslips were mounted with ProLong Diamond Antifade Mountan (Thermo Fisher) and were observed and Z-stack images were acquired by a confocal microscope (FV3000, Olympus) with a 40× and 60× oil objective (UPLXAPO40XO, UPLXAPO60XOHR).

### Western Blotting

BMDMs and HeLa-RIPK3 cells were lysed in extraction buffer (20 mM HEPES [pH 7.4], 150 mM NaCl, 12.5 mM β-glycerophosphate, 1.5 mM MgCl_2_, 2 mM EGTA, 10 mM NaF, 2 mM DTT, 1 mM Na_3_VO_4_, 1 mM PMSF, 20 µg/ml aprotinin, 0.5% Triton X-100). For fractionation of cell proteins, BMDMs were processed with the Qproteome Mitochondrial Isolation Kit (QIAGEN), and the cytosol and the mitochondrial fractions were subjected to Western blotting. Proteins with and without a reducing agent 2-mercaptoethanol were resolved using sodium dodecyl sulphate (SDS)–polyacrylamide gel electrophoresis, and were transferred to polyvinylidene difluoride membranes (Thermo Fisher Scientific). Anti-TAK1 (1:1000), anti-CASP8 (1:1000), anti-MLKL (1:1000), anti-P-MLKL (1:1000), anti-GSDMD (1:1000), anti-GSDME (1:1000), anti-TOM20 (1:1000), anti-GAPDH (1:10,000), and anti-β-actin (1:10,000) were used, and the bound antibodies were visualized with horseradish peroxidase–conjugated antibodies by Clarity and Clarity Max chemiluminescence assay (Bio-Rad).

### Bioenergetics measurements and mitochondrial membrane potential

BMDMs (1 × 10^5^ cells/well) were seeded on a Seahorse cell culture plate. The assay medium was Agilent Seahorse XF Base Medium supplemented with 10 mM glucose, 2 mM glutamine and 1 mM sodium pyruvate and manually adjusted to pH 7.4 with NaOH 0.1 N. To measure oxygen consumption rate (OCR), 0.5 µM Oligomycin A (to block ATP synthesis), 1 µM FCCP (to uncouple mitochondria proton pumping) and 1 µM antimycin A + rotenone (to completely block the electron transport chain) were injected sequentially in a Seahorse XFe96 Analyzer (Agilent Technologies). For measuring mitochondrial membrane potential, tetramethylrhodamine ethyl ester (TMRE) (100 nM) at 30 min prior to harvesting cells, and harvested cells suspension was divided into two tubes followed by incubation with 50 µM carbonyl cyanide m-chlorophenyl hydrazone (CCCP) or vehicle for 15 min. After washing cells with PBS, cells were incubated with 30 nM Sytox Green for 30 min. Live (Sytox green-negative) cells were gated, and the mean fluorescence intensities (MFIs) were determined with a flow cytometer (Accuri C6 Plus, BD Bioscience). The MFIs, subtracted by those with CCCP treated cells, are used as relative membrane potential values.

### Statistical analysis

The box and whisker graphs represent the mean (middle line), the 25th and 75th percentiles (box), the minimum and maximal date points (whisker), and all data points acquired from separately processed samples. Differences between experimental groups were assessed for significance by using the one-way ANOVA with Tukey multiple comparisons test or the unpaired Student’s *t* test (two-tailed) with equal distributions as indicated in the figure legends. Each experiments were repeated three times or more.

## Supplementary information

Supplementary file list and figures

Supplementary video 1

Supplementary video 2

Original WB images

## Data Availability

All data generated or analyzed during this study are included in this published article (and its supplementary information files).
