# Supplementary material for: TAK1 inhibition activates pore-forming proteins to block intracellular bacterial growth through modulating mitochondria
Source: Cell Death Dis. 2025 Jun 18;16(1):456. doi: 10.1038/s41419-025-07760-4 (PMC12177065; doi:10.1038/s41419-025-07760-4)
Supplement: Supplementary file 1 — Supplementary file list and figures [file 41419_2025_7760_MOESM1_ESM.pdf]

Supplementary materials

Title: TAK1 inhibition activates pore-forming proteins to block intracellular bacterial growth through modulating mitochondria

Authors: Wilfred Lopez-Perez[1], Roland Gonzalez-Calderon[1], Kazuhito Sai[1], Prashant Rai[2], Jacqueline M. MacStudy[1], Yosuke Sakamachi[1], Cameron Parsons[3], Sophia Kathariou[3], Michael B. Fessler[2], Jun Ninomiya-Tsuji[1*.]

Supplementary figures: Fig. S1, S2, S3, S4, S5 and S6.

Supplementary video 1 and 2

Lopez-Perez et al. Supplementary Fig. S1

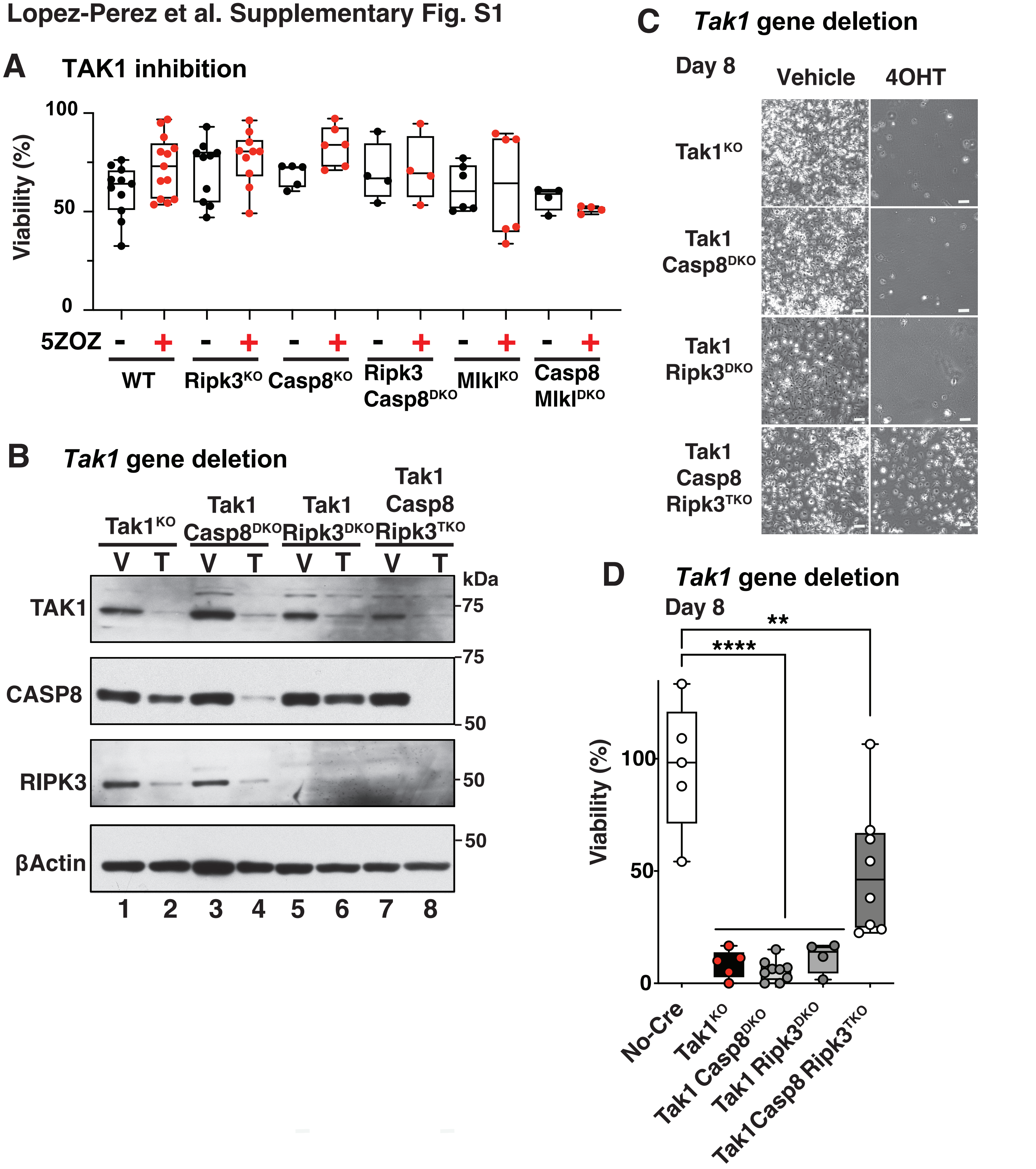

Supplementary Figure S1 ***Casp8*** **and** ***Ripk3*** **double deletion largely rescues** ***Tak1*****-deficient BMDMs**
(A) Cell viability (% of Sytox Green negative cells) at 18 h after 5ZOZ treatment,
(B) Protein levels were analyzed by Western blotting at day 4 post-vehicle (V) or 4OHT (T) treatment.
(C) Representative BMDM photos at 8 days post vehicle or 4OHT treatment are shown. Scale bar, 50 μm
(D) Live cells were quantified by crystal violet assay at day 8. Percentages of 4OHT treated cells relative to vehicle treated cells are shown. One-way ANOVA, multiple comparisons, Tukey test; ****, $p < 0.0001$; ***, $p < 0.001$; **, $p < 0.01$.

Lopez-Perez et al. Supplementary Fig. S2

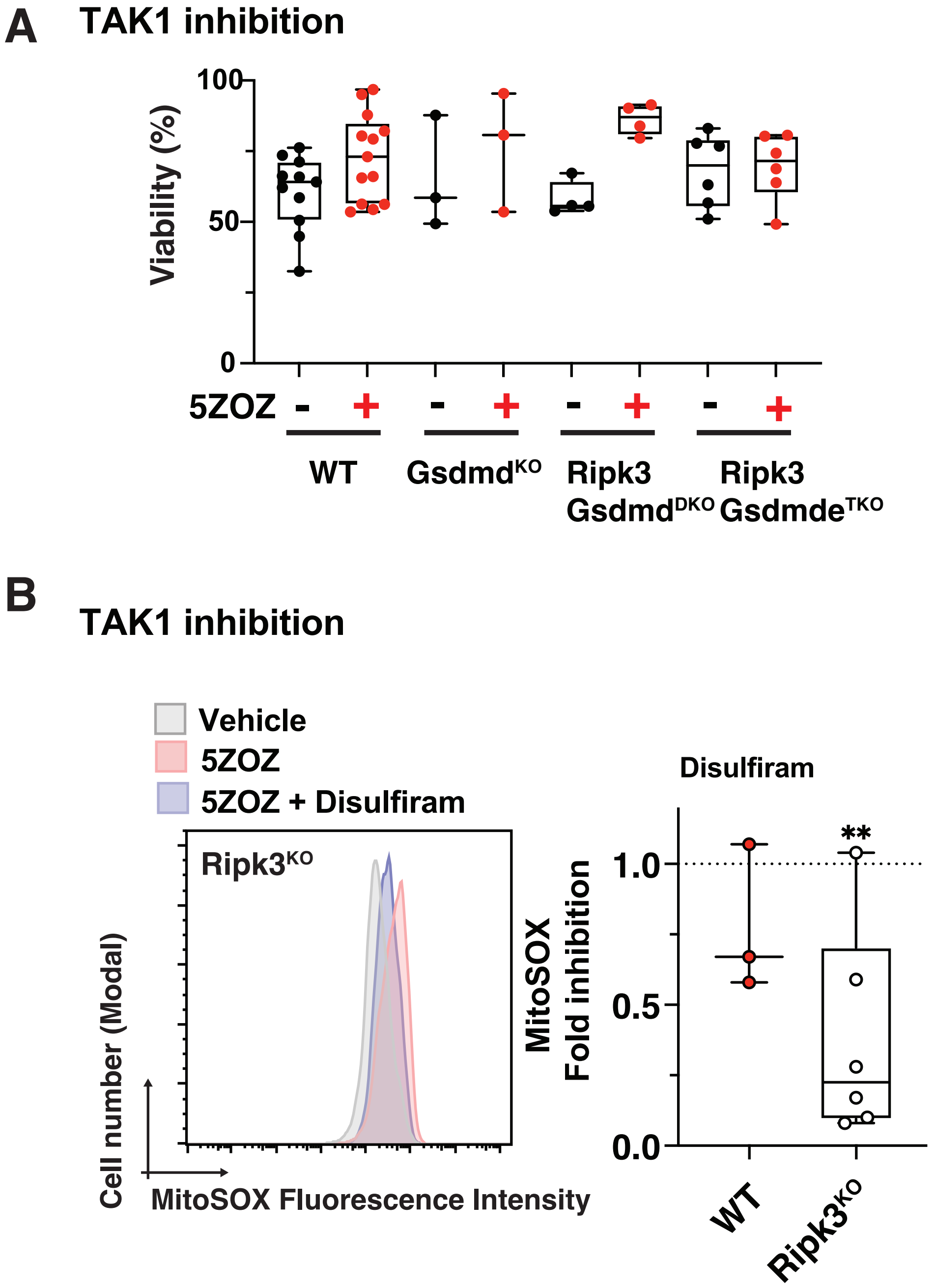

Supplementary Figure S2 Mitochondrial ROS and effects of disulfiram in Salmonella infected BMDMs

(A) Cell viability (% of Sytox Green negative cells) at 18 h after 5ZOZ treatment. The results of WT in Fig.S1A are shown here to provide a baseline comparison.

(B) Wild type and Ripk3-deficient BMDMs were treated 300 nM 5ZOZ alone or together with 50 μM disulfiram (DSF) for 18 h. Representative MitoSOX analysis in Ripk3-deficient BMDMs (left panel) and MFIs in DSF and 5ZOZ co-treated samples relative to 5ZOZ alone-treated controls (right graph) are shown. Students' t-test, **, $p < 0.01$.

Lopez-Perez et al. Supplementary Fig. S3

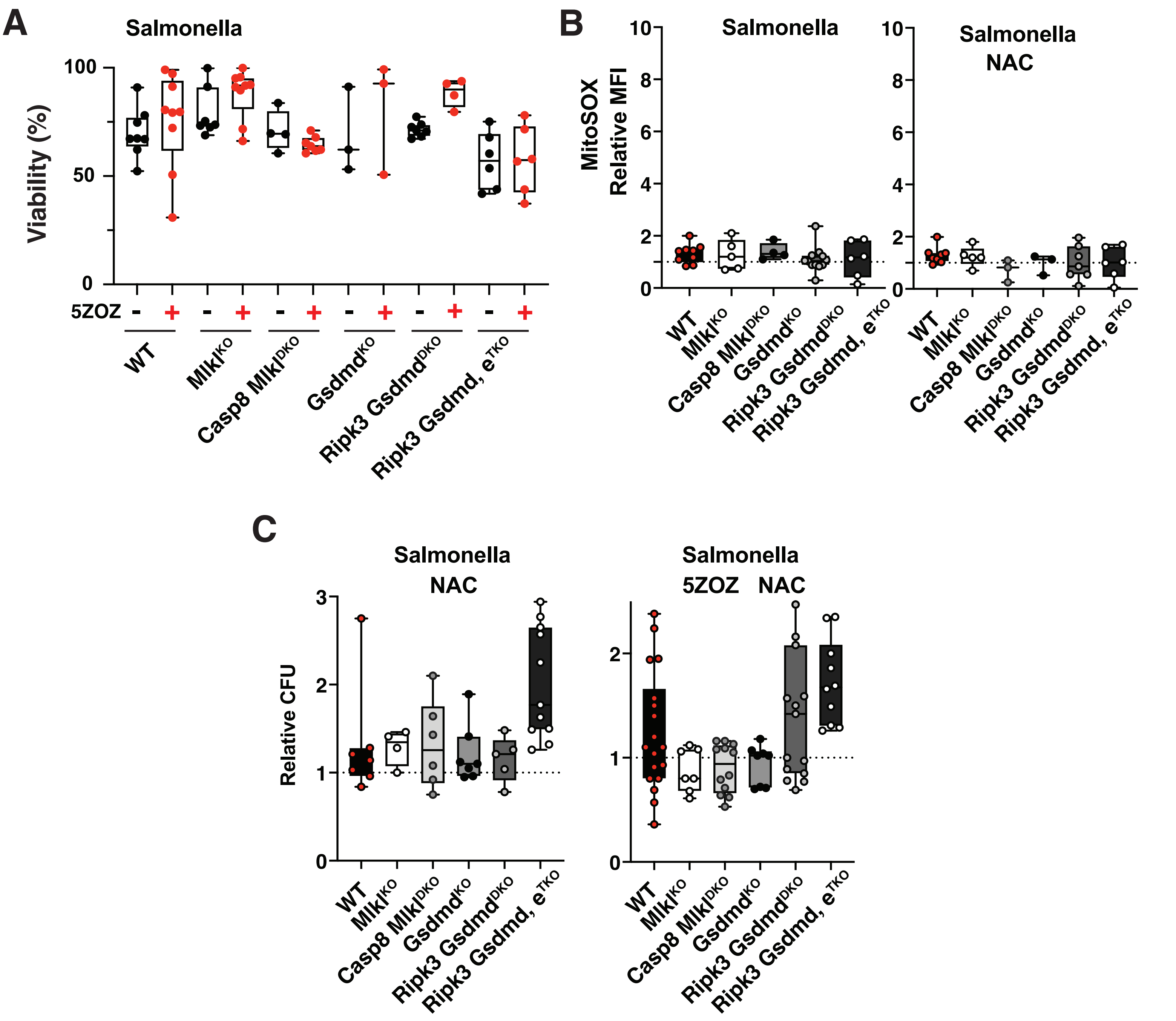

Supplementary Figure S3 Mitochondrial ROS and intracellular Salmonella with and without N-acetyl cysteine

BMDMs isolated from bone marrows with the indicated genotypes were infected with Salmonella (MOI, 10) for 30 min, and extracellular Salmonella were eliminated by gentamicin treatment. BMDMs treated with or without 300 nM 5ZOZ and or 3 mM N-acetyl cysteine (NAC) for 18 h. (A) Cell viablity (% of Sytox Green negative cells). (B) MitoSOX MFI of salmonella infection alon (left) and of salmonella infection with NAC (right) in live BMDMs relative to samples with no-Salmonella infection. (C) Colony numbers of BMDMs treated with NAC alone (left) or 5ZOZ together with NAC (right) relative to salmonella infection alone.

Lopez-Perez et al. Supplementary Fig. S4

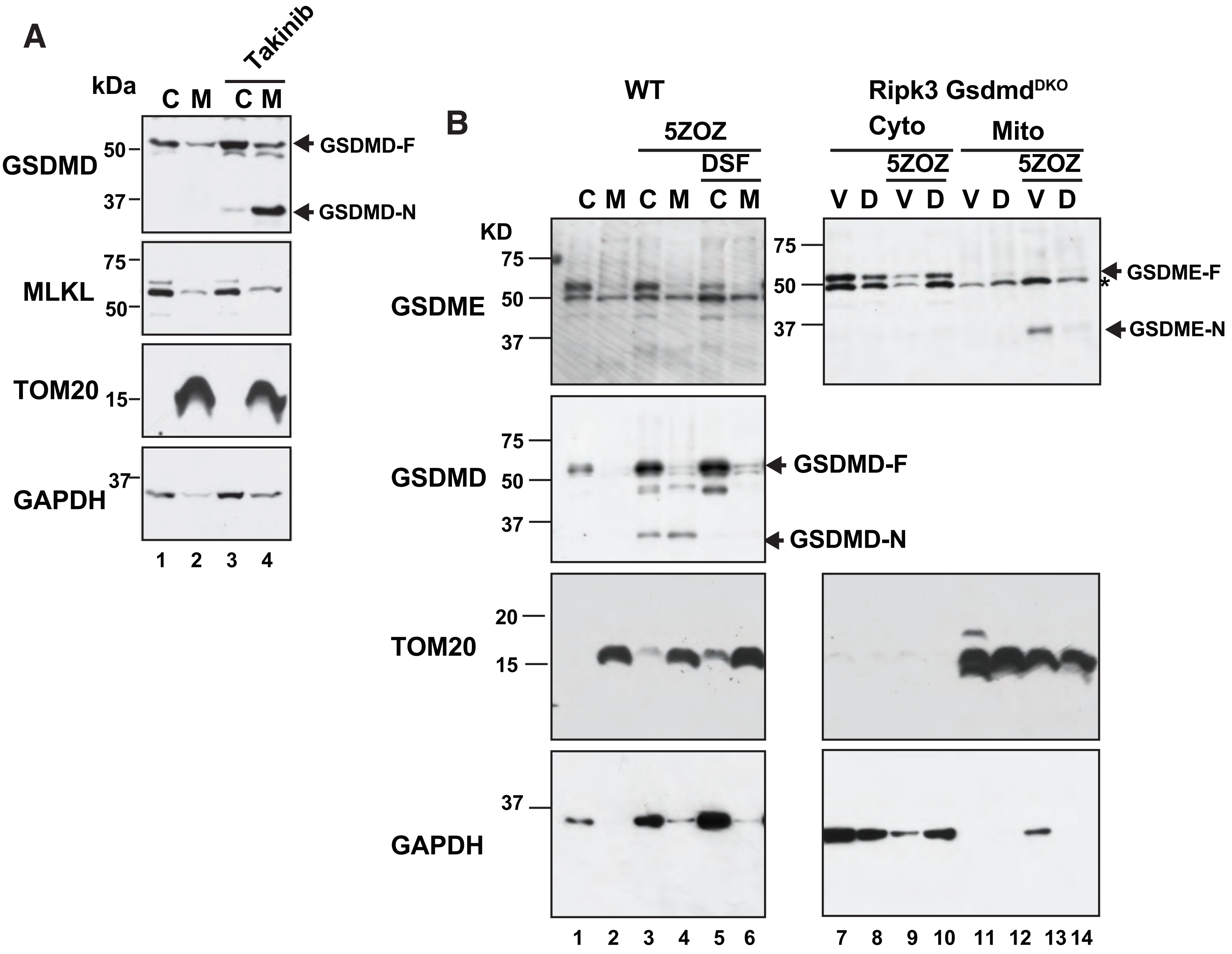

Supplementary Figure S4 Mitochondrial translocation of gasdermins

(A) BMDMs were treated with 300 nM 5ZOZ or 10 μM Takinib for 5 h. (B) Wild type (left panels) and *Ripk3* and *Gsdmd* double-deficient BMDMs were treated with 300 nM 5ZOZ, vehicle (V) or 50 μM disulfiram (DSF or D) for 5 h.
Cell lysates were fractionated into the cytosol (C) and the mitochondria (M). Full length GSDME (GSDME-F), N-terminal GSDME (GSDME-N), full-length GSDMD (GSDMD-F), and N-terminal GSDMD (GSDMD-N) are indicated. Mitochondrial marker TOM20 and cytosolic marker GAPDH are shown as loading controls. *, non-specific band.

Lopez-Perez et al. Supplementary Fig. S5A, B, C

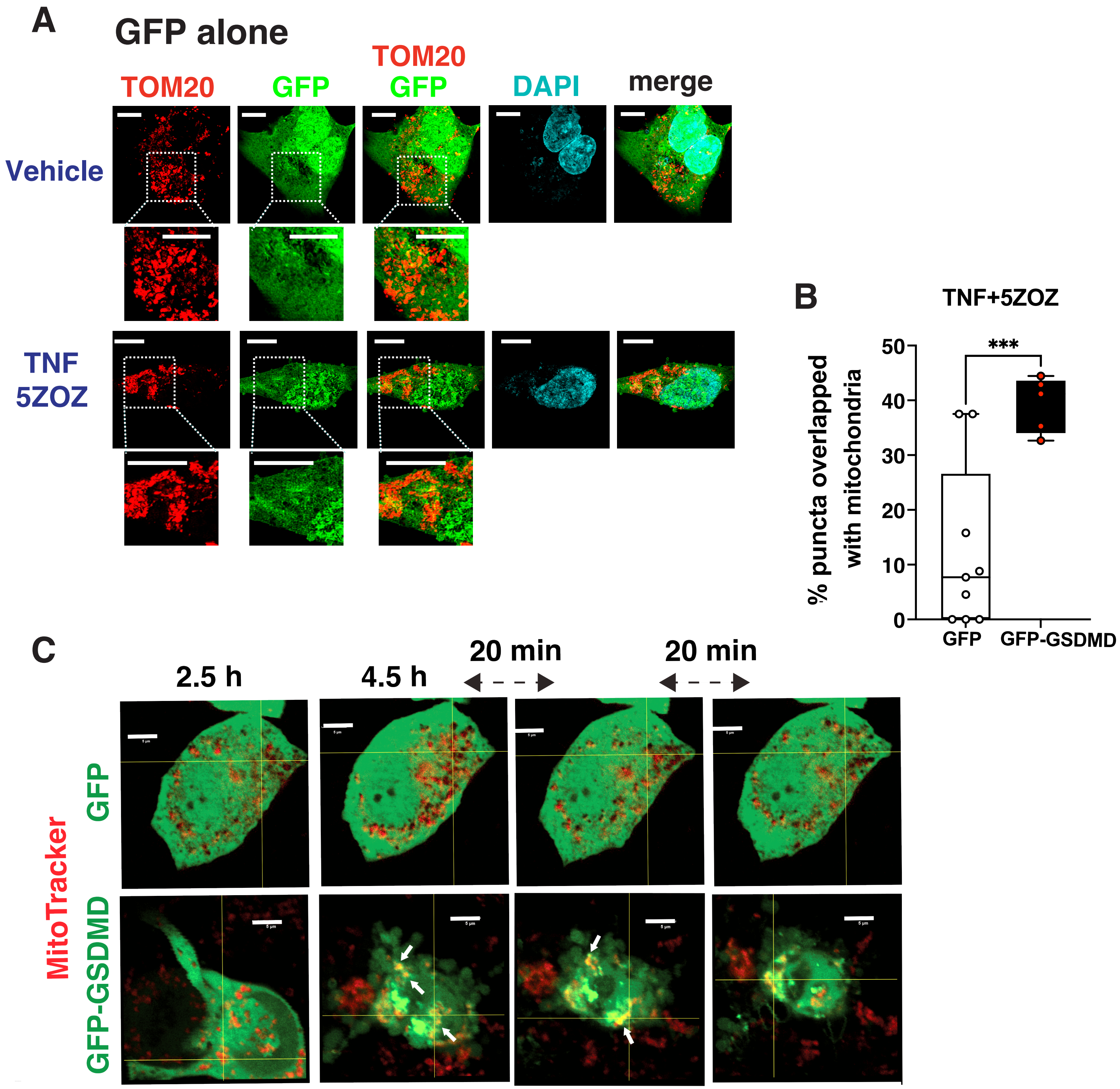

Supplementary Figure S5 GFP-GSDMD is localized to mitochondria
(A) HeLa-RIPK3 cells were transfected with a vector expressing GFP alone as control for the experiments shown in Fig. 5D. At 24 h post-transfection, cells were treated with vehicle or 50 ng/ml TNF and 1 μM 5ZOZ for 5 h. Scale bar, 10 μm. Volumetric (3D) image of the TNF and 5ZOZ treated cell are shown in Supplementary video 2. (B) GFP and GFP-GSDMD puncta overlapped with mitochondria are quantified in the experiments shown in Fig. 5D (GFP-GSDMD) and supplementary Fig.S5A (GFP) with TNF and 5ZOZ treatment. Student t-test, ***, $p < 0.0001$. (C) Time lapse live-cell images with MitoTracker deep red and GFP after 50 ng/ml TNF and 1 μM 5ZOZ treatment. Arrows indicate co-localized GFP-GSDMD with mitochondria. Scale bars, 5 μm.

!

Lopez-Perez et al. Supplementary Fig. S5 D, E, F

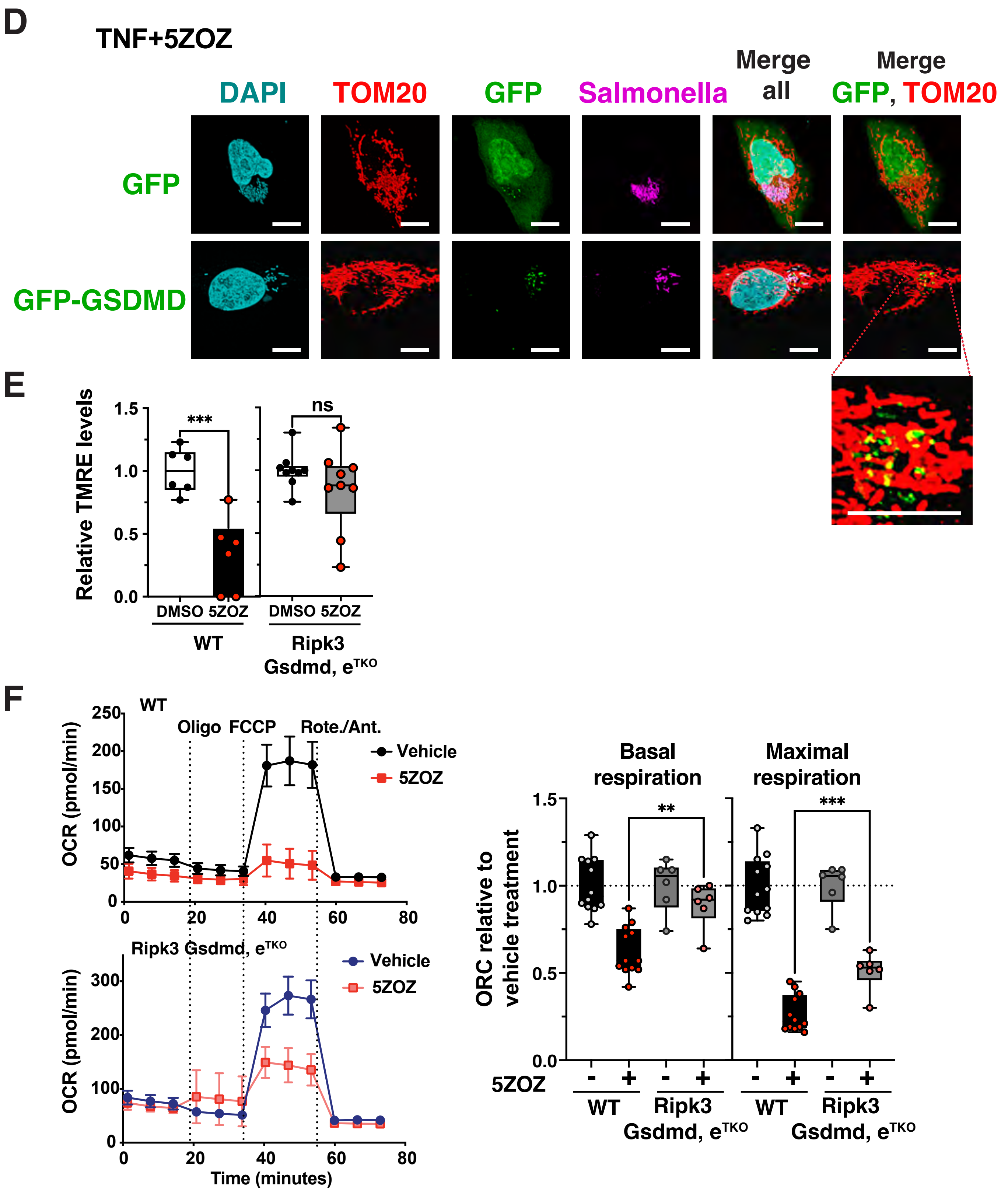

Supplementary Figure S5 GFP-GSDMD localization and mitochondrial respiration
(D) HeLa-RIPK3 cells were transfected with vectors expressing GFP alone or GFP-GSDMD. At 24 h post-transfection, mCherry-Salmonella were infected (MOI100) and extracellular Salmonella were killed by gentamicin. At 24 h post infection, cells were treated with vehicle or 50 ng/ml TNF and 1 µM 5ZOZ, fixed and analyzed with immunofluorescence staining. Scale bars, 10 µm. (E) Mitochondrial membrane potential was determined with TMRE in wild type (WT) and *Ripk3*, *Gsdmd* and *Gsdme* triple deficient (Ripk3, Gsdmd.e TKO) BMDMs with and without 300 nM 5ZOZ (5 h). Student-t test, ***, $p < 0.001$; ns, not significant. (F) OCR was determined in WT and Ripk3, Gsdmd.e TKO BMDMs. OCR relative to vehicle treated cells are shown in the right graph. One-way ANOVA, ***, $p < 0.001$; **, $p < 0.01$.

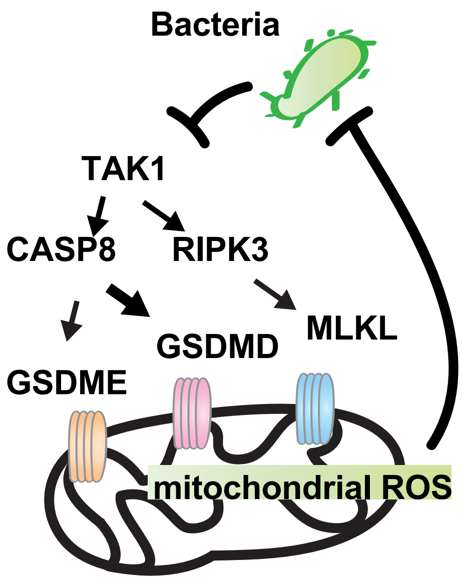

Supplementary Figure S6 Graphic summary
TAK1 inhibition translocates GSDMD and MLKL to mitochondria to promote mitochondrial ROS production. GSDME can play a compensatory role when GSDMD is not available.

Supplementary Video 1
Volumetric images of the cell shown in Fig. 5D, 3rd panel.

Supplementary Video 2
Volumetric images of the cell shown in Supplementary Fig. S5A, 3rd panel.
