# Supplementary material for: TAK1 inhibition activates pore-forming proteins to block intracellular bacterial growth through modulating mitochondria
Source: Cell Death Dis. 2025 Jun 18;16(1):456. doi: 10.1038/s41419-025-07760-4 (PMC12177065; doi:10.1038/s41419-025-07760-4)
Supplement: Supplementary file 4 — Original WB images [file 41419_2025_7760_MOESM4_ESM.pdf]

Fig. 4A

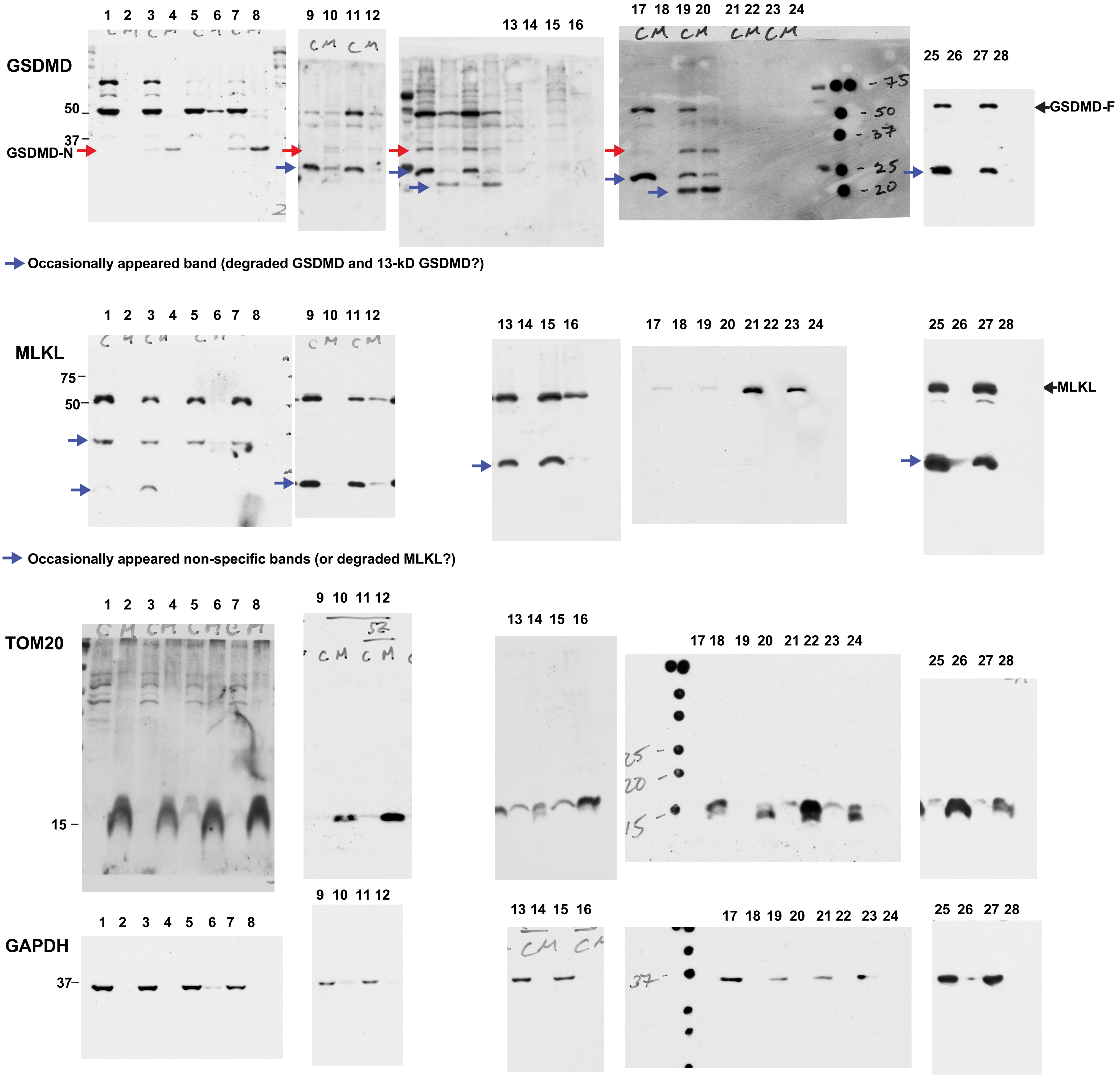
GSDMD
1 2 3 4 5 6 7 8
9 10 11 12
13 14 15 16
17 18 19 20 21 22 23 24
25 26 27 28
50
37
GSDMD-N
GSDMD-F
Occasionally appeared band (degraded GSDMD and 13-kD GSDMD?)
MLKL
75
50
MLKL
Occasionally appeared non-specific bands (or degraded MLKL?)
TOM20
15
GAPDH
37

Fig. 4B

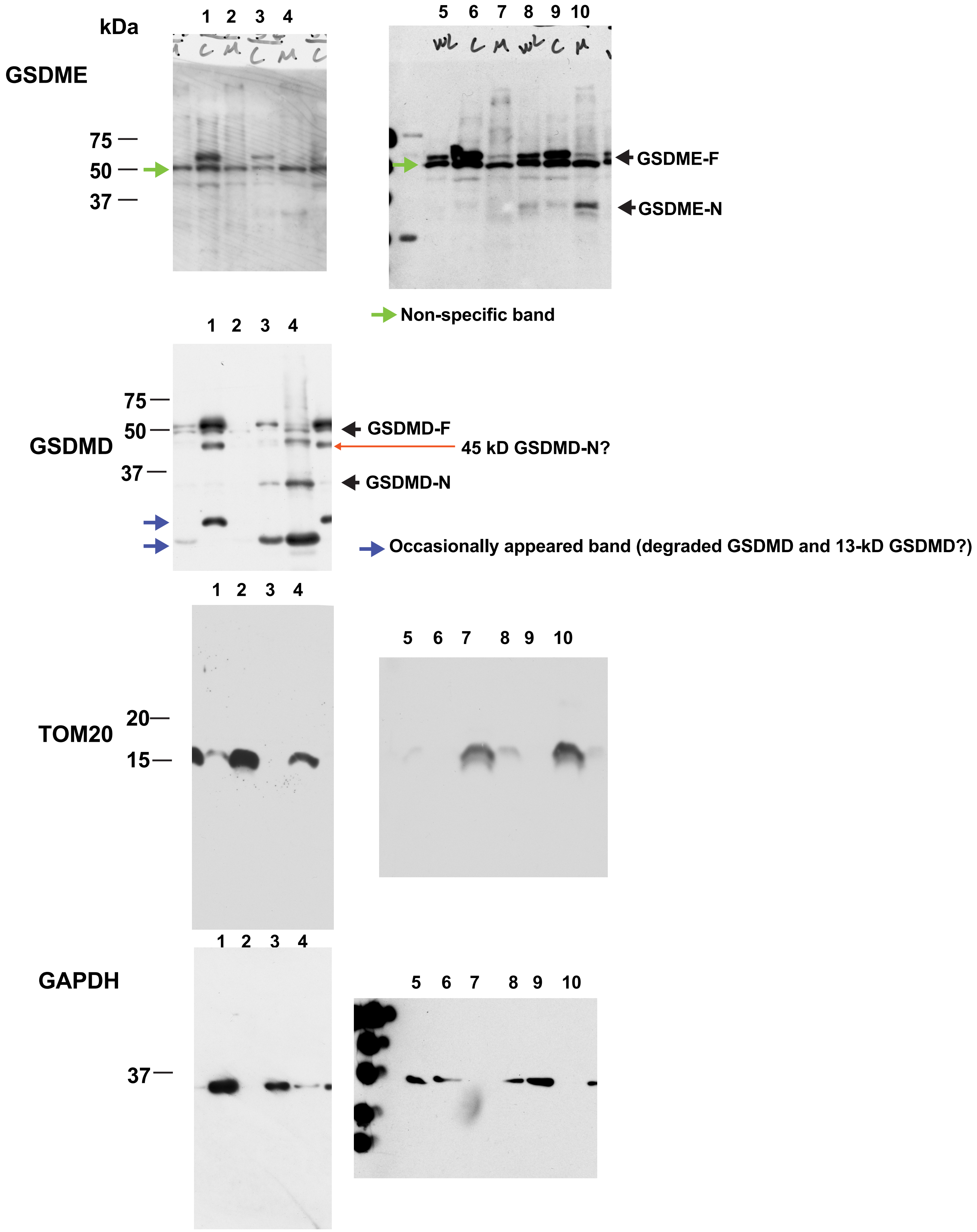

kDa
1 2 3 4
GSDME
75
50
37
5 6 7 8 9 10
GSDME-F
GSDME-N
Non-specific band
1 2 3 4
75
50
GSDMD
37
GSDMD-F
45 kD GSDMD-N?
GSDMD-N
Occasionally appeared band (degraded GSDMD and 13-kD GSDMD?)
1 2 3 4
5 6 7 8 9 10
TOM20
20
15
1 2 3 4
GAPDH
5 6 7 8 9 10
37

Fig. 5A,B

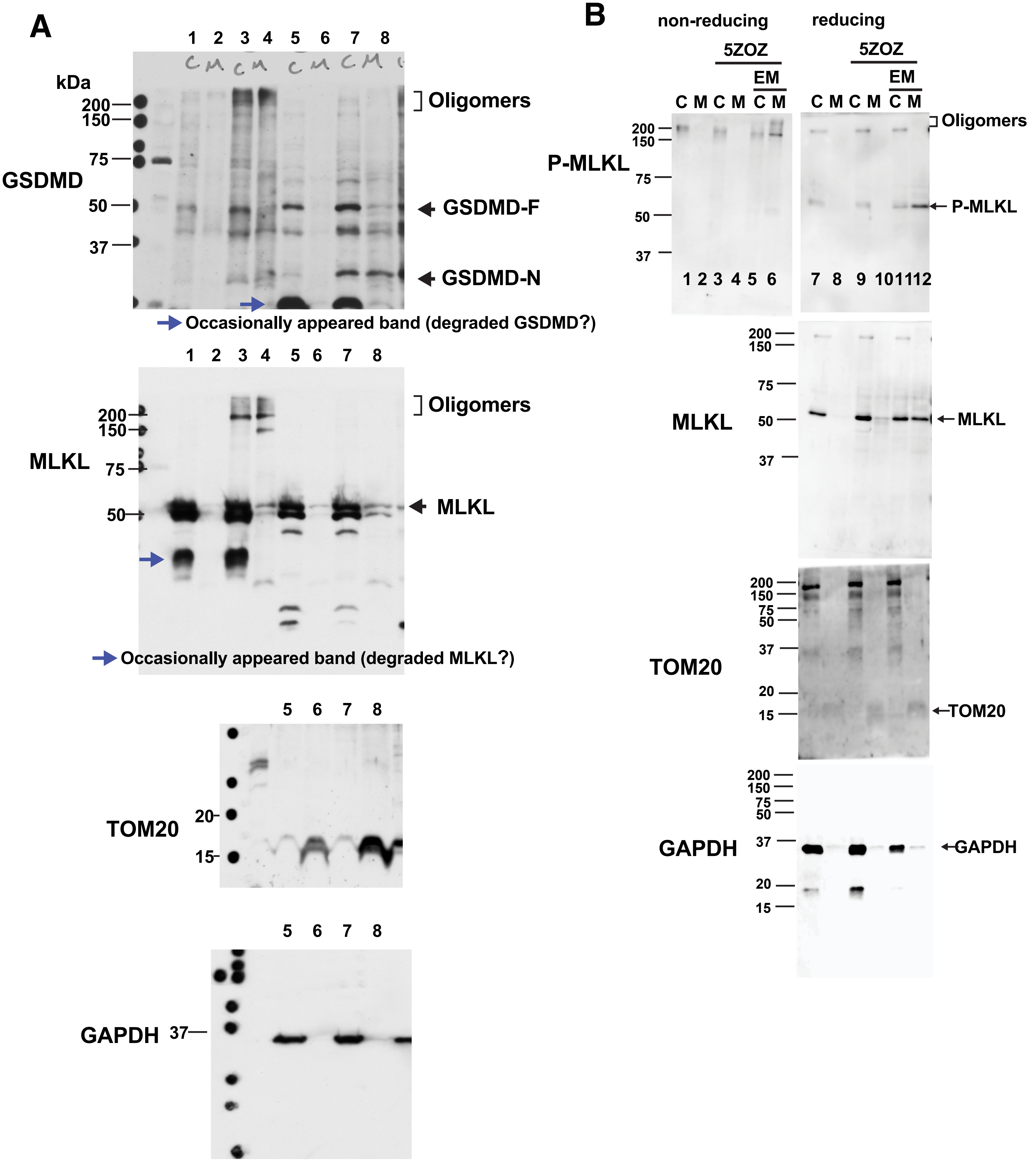
A
1 2 3 4 5 6 7 8
C M C M C M C M
kDa
200
150
75
50
37
GSDMD
Oligomers
GSDMD-F
GSDMD-N
Occasionally appeared band (degraded GSDMD?)
MLKL
Oligomers
MLKL
Occasionally appeared band (degraded MLKL?)
TOM20
20
15
5 6 7 8
GAPDH
37
B
non-reducing
reducing
5ZOZ
EM
C M C M C M
P-MLKL
Oligomers
P-MLKL
1 2 3 4 5 6
7 8 9 10 11 12
MLKL
TOM20
GAPDH

Fig. 5C

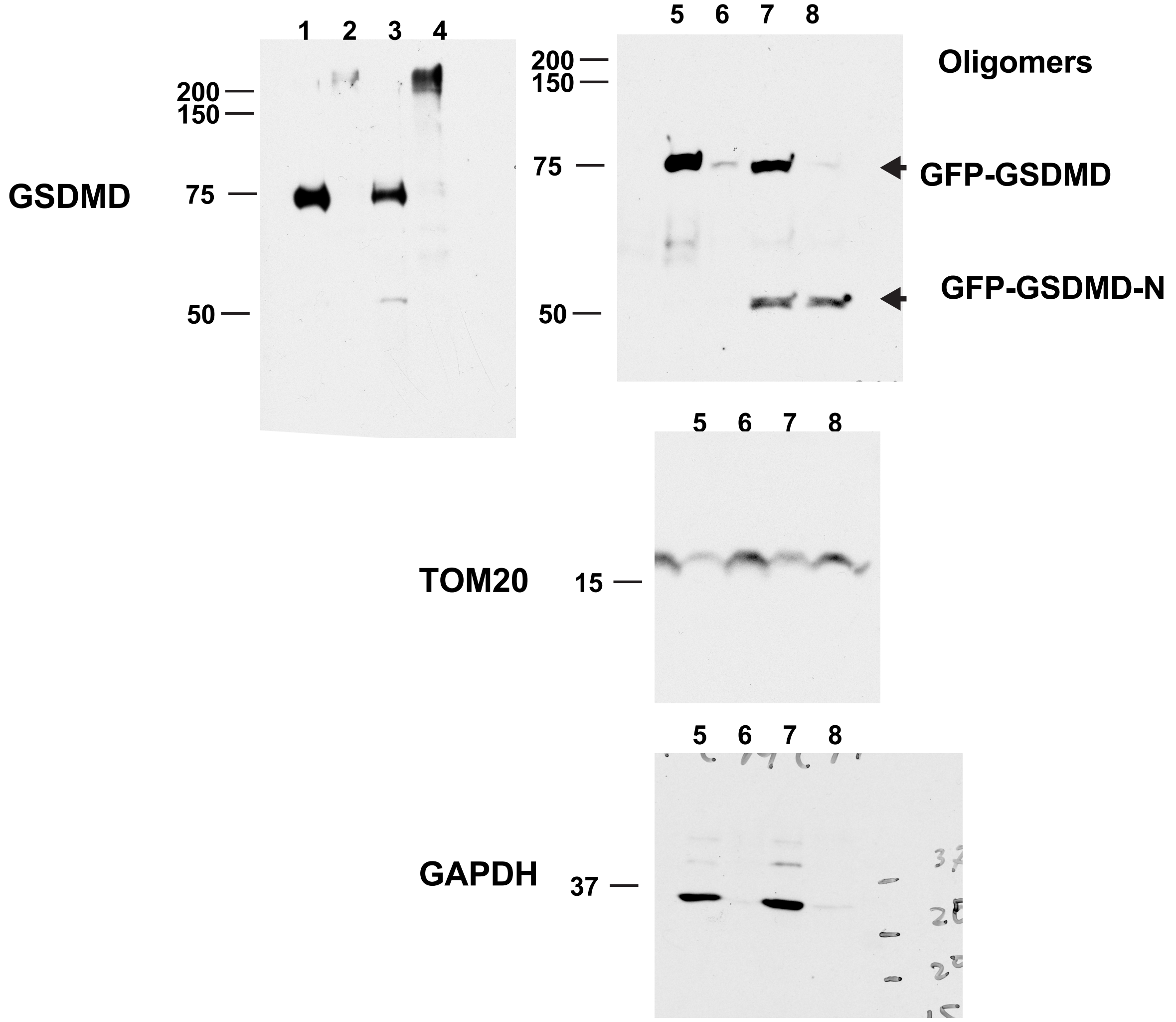
1 2 3 4
200
150
75
50
GSDMD
5 6 7 8
200
150
75
50
Oligomers
GFP-GSDMD
GFP-GSDMD-N
TOM20
15
GAPDH
37

Sup. S1A

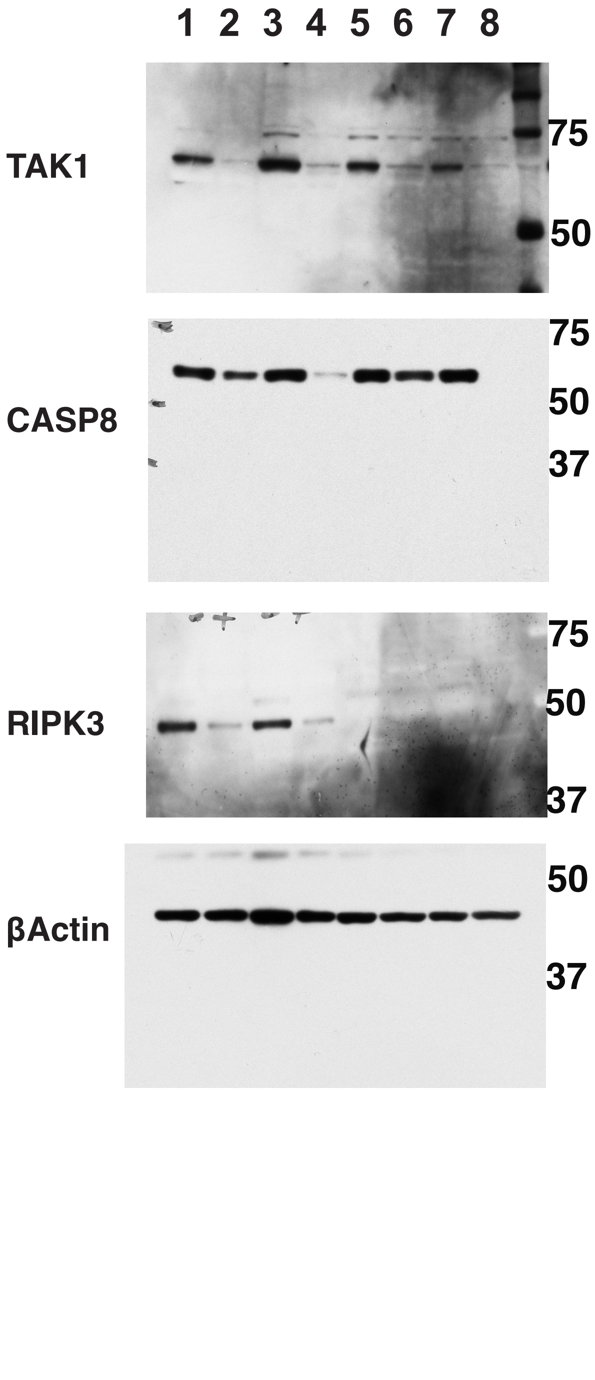

SupFig. S4A

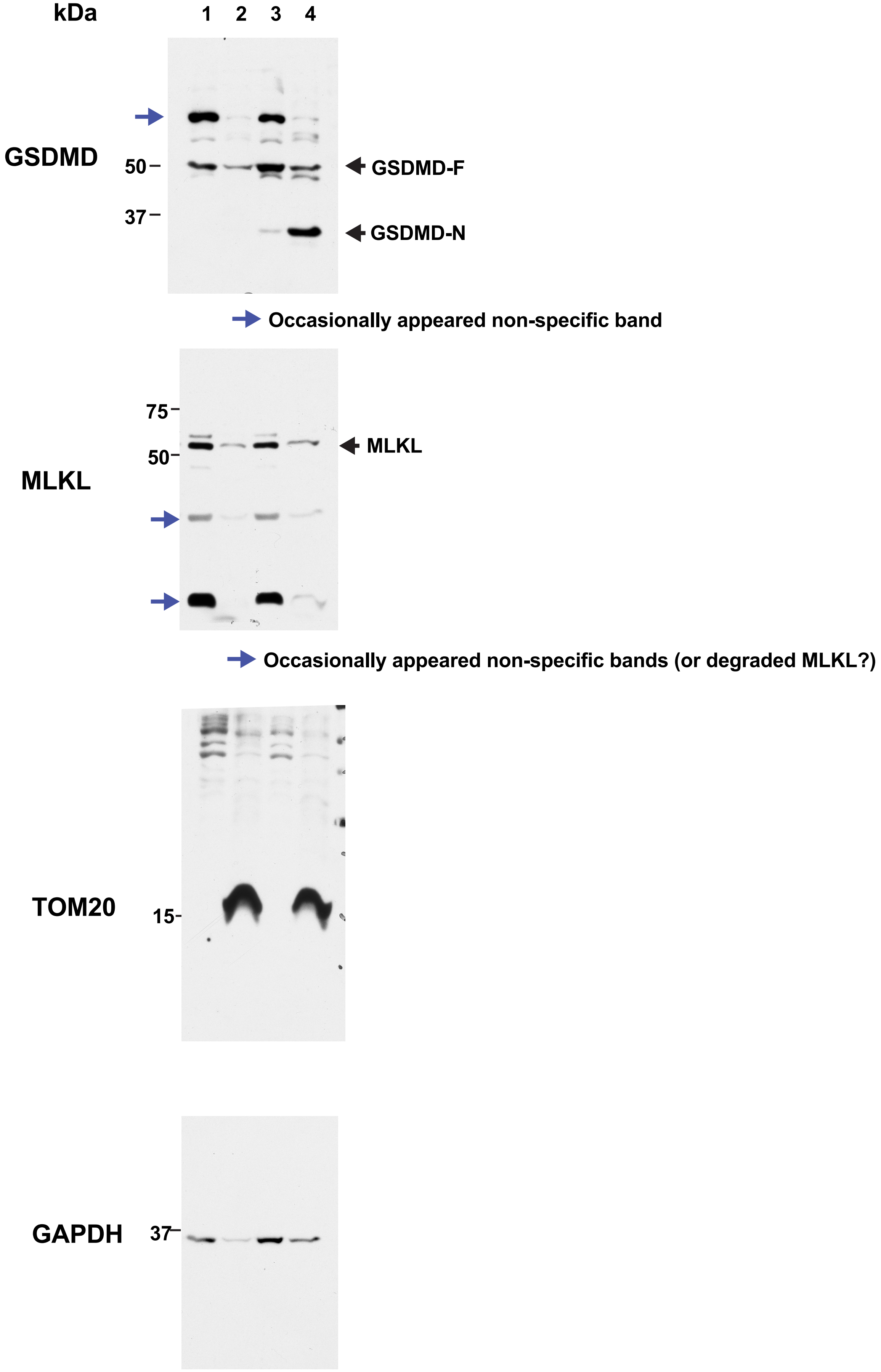
kDa
1 2 3 4
GSDMD
50
37
GSDMD-F
GSDMD-N
Occasionally appeared non-specific band
75
50
MLKL
MLKL
Occasionally appeared non-specific bands (or degraded MLKL?)
TOM20
15
GAPDH
37

SupFig. S4B

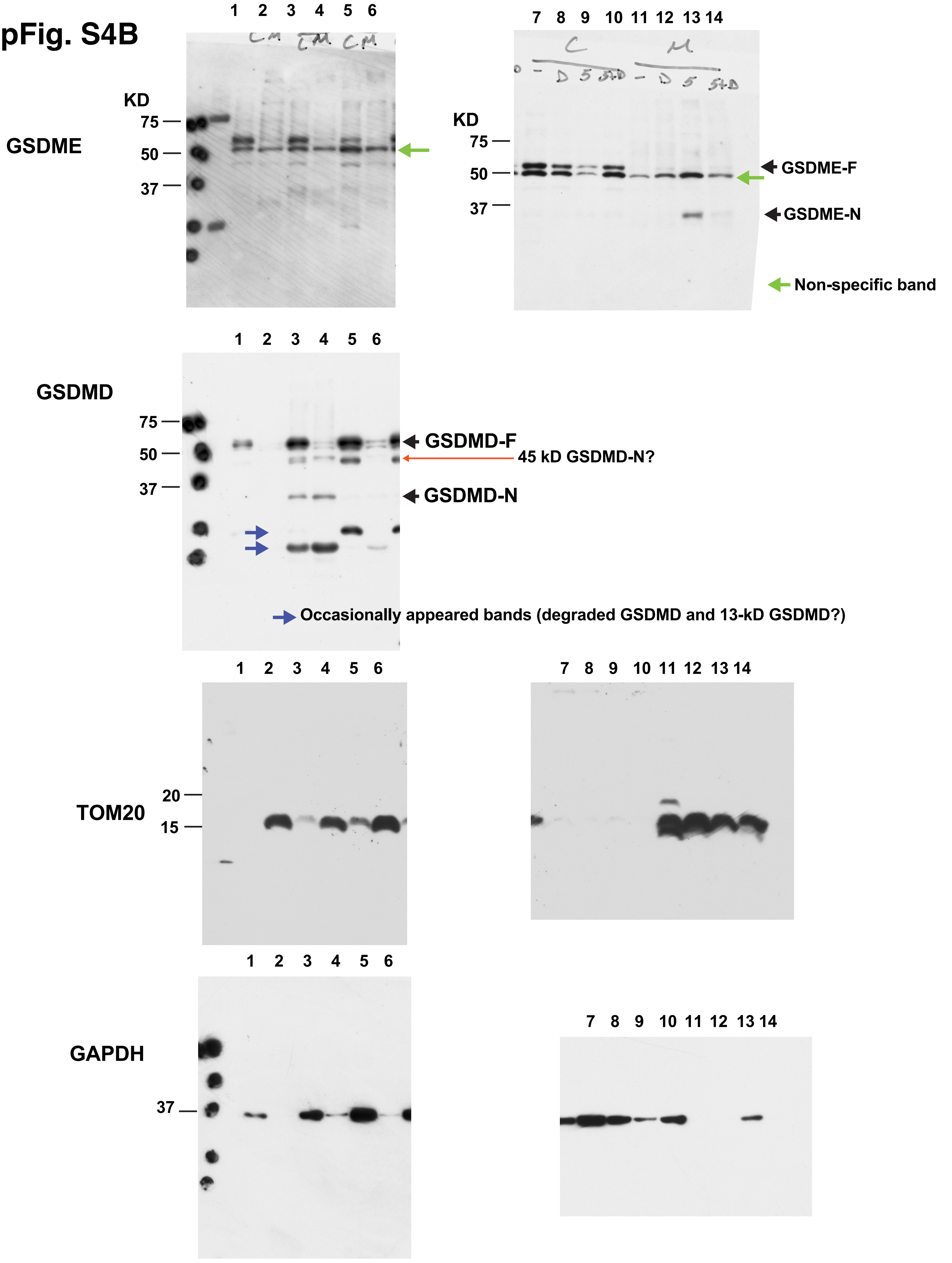
GSDME
KD
75
50
37
1 2 3 4 5 6
7 8 9 10 11 12 13 14
GSDME-F
GSDME-N
Non-specific band
GSDMD
GSDMD-F
45 kD GSDMD-N?
GSDMD-N
Occasionally appeared bands (degraded GSDMD and 13-kD GSDMD?)
TOM20
20
15
GAPDH
37
